# Homotypic cell cannibalism, a cell-death process regulated by the nuclear protein 1, opposes to metastasis in pancreatic cancer

**DOI:** 10.1002/emmm.201201255

**Published:** 2012-07-23

**Authors:** Carla E Cano, María José Sandí, Tewfik Hamidi, Ezequiel L Calvo, Olivier Turrini, Laurent Bartholin, Céline Loncle, Véronique Secq, Stéphane Garcia, Gwen Lomberk, Guido Kroemer, Raul Urrutia, Juan L Iovanna

**Affiliations:** 1 INSERM U1068, CRCM, Cell StressMarseille, France; 2 Institut Paoli-CalmettesMarseille, France; 3 Aix-Marseille UniversityMarseille, France; 4 CNRS, UMR7258, CRCMMarseille, France; 5 Molecular Endocrinology and Oncology Research Center, CHUL Research CenterQuebec, Canada; 6 INSERM U1052Lyon, France; 7 CNRS UMR 5286Lyon, France; 8 Centre de Recherche en Cancérologie de Lyon, Centre Léon BérardLyon, France; 9 University of LyonLyon, France; 10 Laboratory of Epigenetics and Chromatin Dynamics, Gastroenterology Research Unit, Departments of Biochemistry and Molecular Biology, Biophysics, and Medicine, Mayo ClinicRochester, USA; 11 INSERM, U848Villejuif, France; 12 Metabolomics Platform, Institut Gustave RoussyVillejuif, France; 13 Centre de Recherche des CordeliersParis, France; 14 Pôle de Biologie, Hôpital Européen Georges Pompidou, AP-HPParis, France; 15 Université Paris DescartesParis 5, Paris, France

**Keywords:** cell cannibalism, metastasis, Nupr1, pancreatic cancer, TGFβ

## Abstract

Pancreatic adenocarcinoma (PDAC) is an extremely deadly disease for which all treatments available have failed to improve life expectancy significantly. This may be explained by the high metastatic potential of PDAC cells, which results from their dedifferentiation towards a mesenchymal phenotype. Some PDAC present cell-in-cell structures whose origin and significance are currently unknown. We show here that cell-in-cells form after homotypic cell cannibalism (HoCC). We found PDAC patients whose tumours display HoCC develop less metastasis than those without. *In vitro*, HoCC was promoted by inactivation of the nuclear protein 1 (Nupr1), and was enhanced by treatment with transforming growth factor β. HoCC ends with death of PDAC cells, consistent with a metastasis suppressor role for this phenomenon. Hence, our data indicates a protective role for HoCC in PDAC and identifies Nupr1 as a molecular regulator of this process.

## INTRODUCTION

Pancreatic cancer is the fifth leading cause of cancer death in western countries, with a dismal prognosis of less than 5% survival after 5 years and a median survival of less than 6 months after diagnosis. Pancreatic ductal adenocarcinoma (PDAC) is the most common type of pancreatic cancer, representing more than 85% of reported cases. Nowadays, this disease remains incurable, owing to the failure of all currently available treatments, including surgical resection and chemotherapy, to substantially improve patients' survival. It is currently accepted that advances in basic science, by providing knowledge on new pathways that can be therapeutically targeted is the best way to achieve the goal of improving the quality of life, achieve 5 years curative interventions, and to initiate effective chemoprevention program to reduce the burden of this disease.

Human pancreatic tumours display a heterogeneous morphology composed by a non-malignant dense stroma and an infiltrating neoplastic epithelium. In most cases, malignant cells appear organized in well-defined tubular structures that progressively derive into fibroblast-like cell regions of invasive carcinoma. Dedifferentiation of malignant cells is achieved through the epithelial-to-mesenchymal transition (EMT), a crucial milestone of tumour development and metastasis triggered by the transforming growth factor β (TGFβ) (Cano et al, 2010; Thiery et al, 2009; Truty & Urrutia, 2007). In addition, variations of PDAC pathophysiology occur in a number of cases. For many years, pathologists have observed, and unfortunately not systematically reported, cases displaying dedifferentiated PDAC cells with atypical morphology characterized by enlarged size and prominent mucinous vacuoles that contained other cells (Silverman et al, 1988; Tracey et al, 1984). The process that allows the formation of such PDAC cell-in-cell structures and their fate had remained elusive. Similar cell-in-cells have been observed in other malignancies for over a 100 years (Steinhaus, 1891; Stroebe, 1892), and various functions have been predicted for this phenomenon, including immune-suppression through heterotypic engulfment and killing of immune cells or cancer cell feeding upon starvation (Caruso et al, 2002; Fais, 2007; Lugini et al, 2006; Wang et al, 2009). Hence, a pro-tumoural function has been foreseen for cell cannibalism. On the other hand, cell-in-cells were recently reported to arise from breast cancer cells that invade each other *in vitro* leading to a cell-death process called entosis, for which an eventual tumour suppressive role was proposed (Overholtzer et al, 2007). Although in both cases cell-in-cell figures are formed, entosis is a homotypic cell-in-cell phenomenon while cannibalism can be either homogeneous or heterogeneous and implicates engulfment of living or dead cells. To date, whether cell-in-cells in PDAC result from cell cannibalism, and their impact in patients' prognosis remain to be determined. Moreover, the molecular pathways associated to this phenomenon in PDAC need to be elucidated.

In order to shed light into these questions, we performed an in depth characterization of cell-in-cell structures in human PDAC and we searched for an eventual association between these structures and the clinicopathological history of the corresponding patients. Based on results obtained from the characterization of cell-in-cells in human PDAC samples, we analyzed the putative role of the TGFβ-induced chromatin factor nuclear protein 1 (Nupr1) in the formation of these structures. Nupr1, also known as p8 or candidate of metastasis-1 (Com-1) (Bratland et al, 2000; Mallo et al, 1997; Vasseur et al, 1999), is a basic helix-loop-helix transcription co-factor strongly induced by stress (for review, Cano & Iovanna, 2010) and upon stimulation by TGFβ (Garcia-Montero et al, 2001), which was associated to metastasis potential of breast cancer cells (Ree et al, 1999). Interestingly, Nupr1 is overexpressed in late stages of PDAC and their metastases (Ito et al, 2005; Su et al, 2001a, b), is involved in resistance to gemcitabine (which is the most widely used chemotherapy against PDAC (Giroux et al, 2006)), and its expression was associated to poor prognosis in patients with PDAC (Hamidi et al, 2012). In this study, we used cells and tissues of human and mouse origin to perform an extensive series of cellular, biochemical, and molecular studies that allowed us to demonstrate that inactivation of Nupr1 provokes a genetic reprogramming in PDAC cells that elicits homotypic cell cannibalism (HoCC)-associated cell-death. Furthermore, we show that TGFβ stimulation enhances HoCC in Nupr1-depleted cells and we show evidence for the implication of Nupr1 in TGFβ-induced EMT. Finally, we discuss the Nupr1-based molecular relationship between HoCC and metastasis and its potential use for anticancer therapy.

## RESULTS

### Human pancreatic adenocarcinomas display discrete regions containing atypic cell-in-cell structures

**Figure 1:**
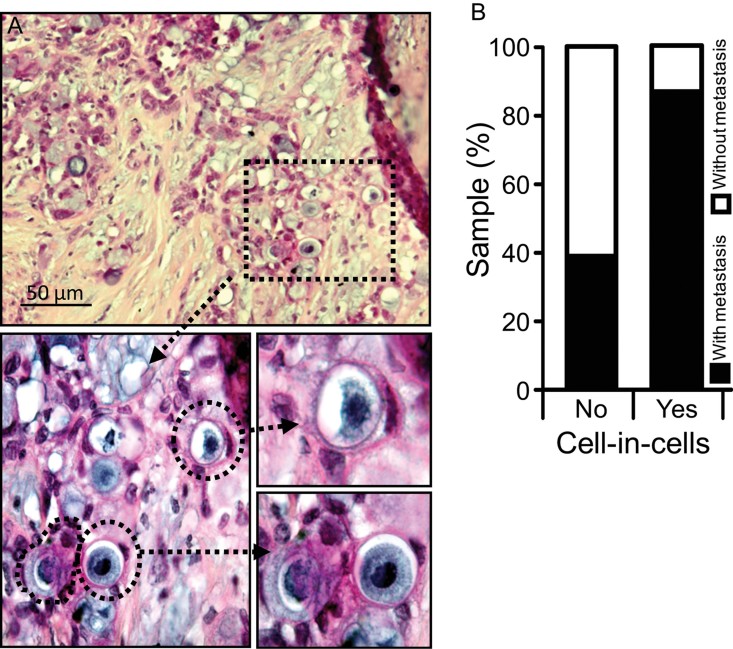
Cell cannibalism in human pancreatic adenocarcinoma H&E staining of human invasive pancreatic adenocarcinoma presenting with cannibal cell-in-cells.Histogram shows proportions of metastasis-free and metastasis-bearing PDAC patients within our cohort.

The current study originated from the histological observation that human pancreatic tumours display undifferentiated cancer tissue areas containing a pool of cancer cells with atypical characteristics, namely, the ability to form cell-in-cell bodies indicative of cell engulfment or cannibalism. We sought to determine the frequency of these events in human pancreatic invasive adenocarcinomas and their impact on patients' prognosis. Therefore, we searched for cell-in-cell events within 36 human PDAC specimens obtained after surgical resection from a cohort of patients with available clinical history. Of note, patients within our cohort were metastasis-free at the time of surgery. After careful histological analysis, we found that thirteen PDAC specimens from our cohort displayed discrete regions (corresponding to 1–10% of the analyzed tumour area) containing cell-in-cell figures that evoked cancer cell cannibalism, which appeared at a frequency of 3.5 ± 0.8% (Fig 1A). Next, we searched for an eventual correlation between the presence of cell-in-cells and the clinicopathological features of the patients, including age, gender, post-operatory survival and the development of metastasis (Supporting Information Table S1). Importantly, we found that only two out of thirteen patients displaying ‘cannibal’ cell-in-cell structures developed metastasis (Fig 1B), whereas fourteen out of twenty-three patients without cell-in-cells did develop metastasis (*X*^2^ = 6.34; *p* = 0.0118) indicating an inverse relationship between cannibalism and metastasis and suggesting an anti-metastasis role of cell-in-cell structures.

### PDAC cell-in-cells undergo cell death, display both epithelial and phagocyte markers but lack Nupr1 expression

**Figure 2:**
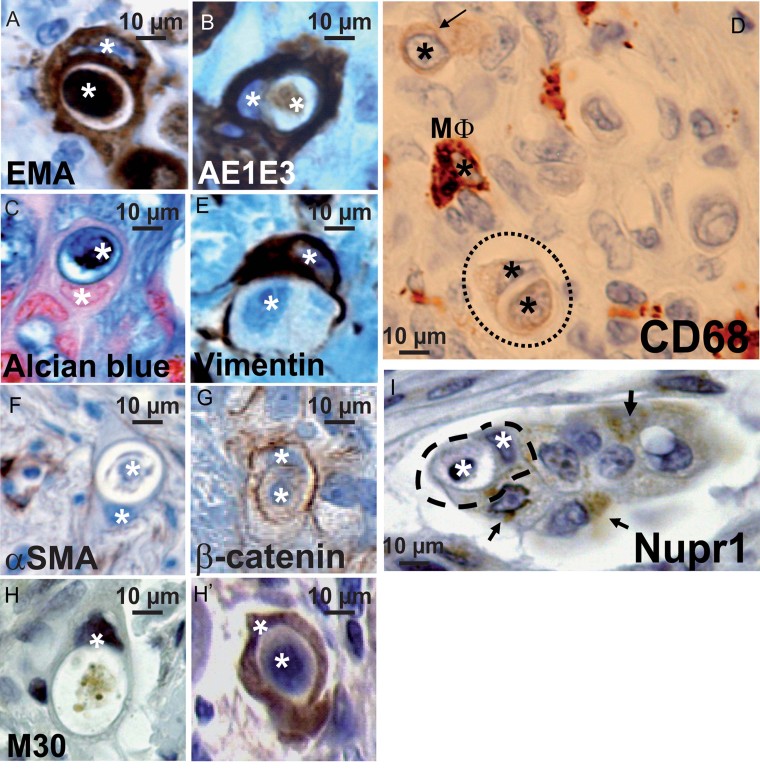
Human pancreatic adenocarcinoma cell-in-cells display markers of epithelial and phagocytoid cells, but lack Nupr1 expression **A-H'.** Immunohistochemical staining of epithelial markers EMA (**A**) and AE1E3 (**B**), the phagocytic marker CD68 (**D**), the fibroblast markers vimentin (**E**) and αSMA (**F**), β-catenin (**G**), caspase-cleaved cytokeratin-18 (stained by the M30 antibody, **H** and **H′**), and Nupr1 (**I**). In (**C**), alcian blue staining shows the mucinous character of cannibal vacuoles. In (**D**) and (**I**), arrows indicate CD68-dull and Nupr1-expressing PDAC cells, respectively. Asterisks mark nuclei. Mφ = macrophage.

In order to characterize the nature of the presumable ‘cannibal’ and ‘prey’ cells forming cell-in-cells, we performed immunohistochemical epithelial membrane antigen (EMA) and AE1E3 staining that confirmed their epithelial origin (Fig 2A and B). Vacuoles of cannibal cells were filled with mucus as shown by strong alcian blue staining (Fig 2C). Interestingly, the epithelial cancer cell-in-cells also displayed an ectopic expression of the macrophage marker CD68 (Fig 2D), which was lower compared to macrophages (see Mφ in Fig 2D). Similarly, cannibal cells were positive for vimentin, a mesenchymal marker known to arise from TGFβ-induced EMT, although the absence of α-smooth muscle actine (αSMA) and the membrane β-catenin localization indicated that cannibal cells did not achieve the classical EMT (Fig 2E–G). Caspase-cleaved cytokeratin-18 staining (revealed by the M30 Cytodeath^©^ antibody) showed that both the inner and outer cells, undergo cell death (Fig 2H and H'). More importantly, cannibal cells lacked the expression of the chromatin protein Nupr1 (Fig 2I and Supporting Information Fig S1), in contrast to glandular epithelial cells that displayed distinct positive Nupr1 staining (Supporting Information Fig S1), suggesting that this chromatin regulator may have an antagonist effect on cannibalism. Thus, initial data combining careful histopathological analysis and use of defined cell lineage markers, suggested that PDAC epithelial cells may acquire a phagocyte-like phenotype characterized by the ectopic expression of CD68 and the ability to cannibalize, a feature that has previously remained unexplored. In addition, PDAC cannibal cells retain the expression of epithelial markers, suggesting that these cells did not achieve EMT. These observations, along with our long term interest in the role of Nupr1 in pancreatic cancer, led us to test the hypothesis that low levels of Nupr1 may elicit a phagocyte-like phenotype that results in cell cannibalism, while limiting EMT in PDAC cells.

### Cell cannibalism is repressed by Nupr1 and enhanced by TGFβ-stimulation in PDAC cells

**Figure 3:**
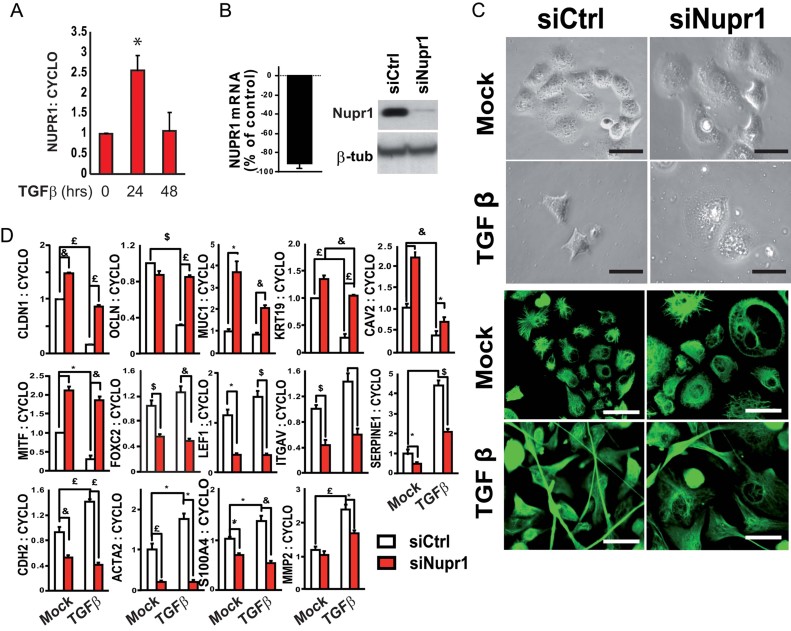
Nupr1-knockdown impairs acquirement of TGFβ-induced EMT features in Panc-1 cells Data are means of triplicates ± SEM. *p ≤ 0.05, $*p* ≤ 0.01, £*p* ≤ 0.005, &*p* ≤ 0.001. qRT-PCR showing Nupr1-expression in mock and TGFβ-treated Panc-1 cells.qRT-PCR (left) and immunoblot (right) showing reduced Nupr1 expression in Panc-1 cells transfected with a Nupr1-specific siRNA (siNupr1) compared to cells transfected with a control siRNA (siCtrl).(Top) Phase contrast microphotographs illustrating alterations in cell morphology and size upon Nupr1-knockdown, before and after 24 h of TGFβ1 stimulation (10 ng/ml). (Bottom) Immunofluorescent detection of vimentin, 72 h after TGFβ1 treatment of Nupr1-depleted or control cells. Note that Panc-1 cells present the particular feature of a strong vimentin expression previous to TGFβ stimulation, although they remain dependent on TGFβ for the induction of fibroblastic markers such as *CDH2*/N-cadherin (see **D**).qRT-PCR showing altered expression of epithelial and fibroblastic genes in Nupr1-depleted cells compared to controls, after 24 h of TGFβ1 treatment. Cyclophilin A mRNA expression was used for normalization.

**Figure 4:**
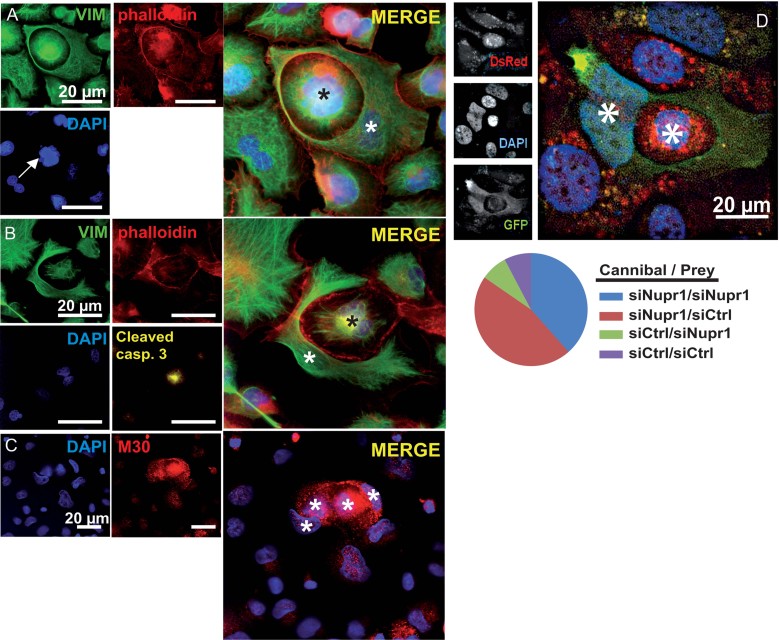
Nupr1-depletion is permissive for TGFβ-stimulated PDAC cell cannibalism and subsequent cell death Fluorescence microscopy pictures of Nupr1-depleted cells after 48 h of TGFβ stimulation. Actin (red as in **A** and **B**) and vimentin (green) immunostaining. Note the nuclear fragmentation of the inner cell (arrow).Cleaved-caspase 3 (yellow) staining.Caspase-cleaved cytokeratin-18 (red) immunostaining revealing cell death of cannibal and prey cells.Nupr1-depleted EGFP-expressing Panc-1 cell were mixed to siCtrl-transfected DsRed-expressing Panc-1 cell before TGFβ treatment; (left) fluorescence microscopy picture showing a green Nupr1-depleted cell cannibalizing a control cell (right). Pie chart showing proportion of cannibal cells (total cannibal cells counted *n* = 39). Asterisks mark nuclei.

**Figure 5:**
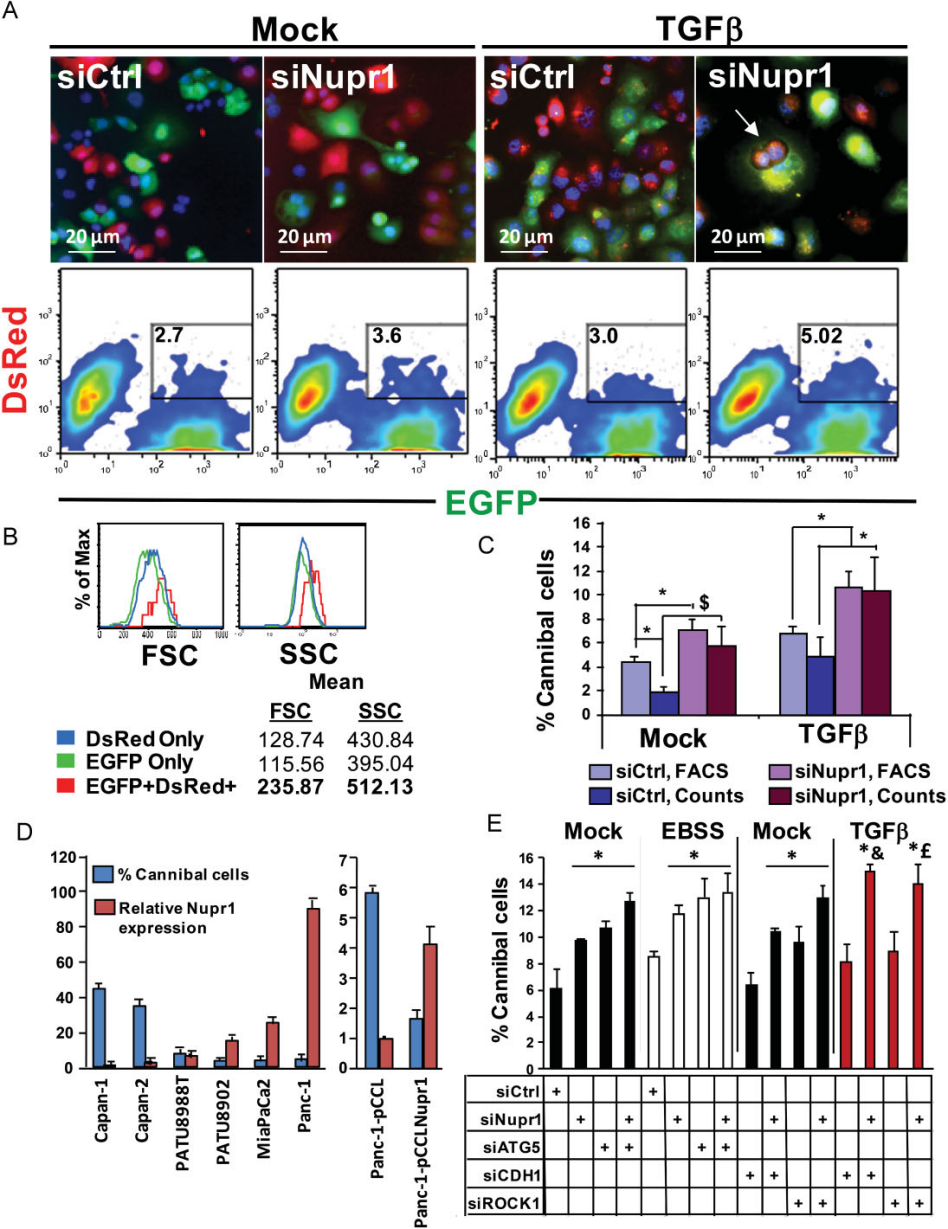
Quantification of cannibalism by FACS EGFP- and DsRed-expressing Panc-1 cells were mixed in equal numbers and transfected with Nupr1-specific (siNupr1) or control (siCtrl) siRNAs before 48 h of TGFβ treatment. FACS-derived dot plots (bottom) and corresponding fluorescent microscopy pictures (top) illustrate the quantification of cell cannibalism.Histograms show mean cell size (FSC) and granularity (SSC) of single (GFP only and DsRed only) and cannibal (GFP^+^DsRed^+^) TGFβ-treated (48 h) Nupr1-depleted cells as measured by flow cytometry and after exclusion of cell debris.Histogram shows the microscopic and FACS-based quantification of cannibalism. FACS data are mean of triplicates ±SEM. *p ≤ 0.05, $*p* ≤ 0.01.Histogram showing the relative Nupr1 expression (red) in pancreatic cancer cell lines Capan-1, Capan-2, PATU8988T, PATU8902, MiaPaCa2 and Panc-1 compared to Capan-1(left), or Panc-1 transduced with pCCL-Nupr1 or empty pCCL lentiviral vectors (right) as measured by qRT-PCR, and the corresponding % cannibal cells measured by FACS for each cell line (blue).Like in (**A**), fluorescent Panc-1 cells were mixed and transfected with the indicated siRNAs, then cultured for 48 h in EBSS medium or in conventional medium with or without TGFβ. Histogram shows the percentage of cannibal cells measured by FACS after 48 h. FACS data are mean of triplicates ± SEM. & and £ are *p* ≤ 0.05 compared to siCtrl, siCDH1 and siROCK1, respectively.

In order to test the hypothesis of an antagonist effect of Nupr1 in PDAC cell cannibalism, we used the human PDAC cell line Panc-1, which strongly expresses Nupr1 (Giroux et al, 2006; Malicet et al, 2003) and investigated whether Nupr1-inactivation would elicit cell cannibalism and under which conditions. Since PDAC cannibal cells conserved strong expression of epithelial markers, we predicted that these cells responded poorly to TGFβ in terms of EMT triggering. Thus, we knocked-down Nupr1 expression in these cells using a specific small interfering RNA (siRNA) (Fig 3), cultured these cells with or without TGFβ, and searched for the appearance of HoCC *in vitro*. We reported previously that TGFβ-stimulation enhances Nupr1 expression during the acute response (Malicet et al, 2003), which was also the case during the present study (Fig 3A). Congruently with previous reports (Ellenrieder et al, 2001a, b), upon TGFβ treatment and in contrast to Nupr1-depleted cells, control Panc-1 cells acquired several features of EMT (Fig 3C and D) including a spindle-like morphology (as depicted by light microphotographs and vimentin staining), down-regulation of epithelial genes (*i.e. CLDN1* (claudin 1), *OCLN* (occludin), *MUC1* (mucin 1), *KRT19* (keratin 19), *CAV2* (caveolin 2) and *MITF* (microphthalmia-associated transcription factor)) and up-regulation of mesenchymal (*i.e. FOXC2* (forkhead box C2), *LEF1* (lymphoid enhancer-binding factor 1), *ITGAV* (integrin, alpha V), *SERPINE1* (plasminogen activator inhibitor-1)) and fibroblast-related genes (*i.e. CDH2* (N-cadherin), *ACTA2* (actin, alpha 2), *S100A4* (S100 calcium binding protein A4), *MMP2* (matrix metallopeptidase 2)). This suggested that low levels of Nupr1 are inhibitory for TGFβ-induced EMT, thus presenting the first evidences of the involvement of this TGFβ-regulated chromatin protein in EMT. Strikingly, a fraction of Nupr1-depleted cells appeared to cannibalize their siblings upon TGFβ treatment, giving rise to homotypic cell-in-cell bodies (Fig 4A), consistent with the observations of Nupr1-negative cannibal cells in human pancreatic tumours. Similar to phagocytosis and the recently described entosis (Overholtzer et al, 2007), HoCC in PDAC cells was associated to cytoskeleton rearrangement, as shown by the formation of a vimentin- and actin-containing ring around the cannibalized cell. In addition, nuclear fractionation (Fig 4A) and staining of cleaved (activated) caspase 3 and caspase-cleaved cytokeratin-18 (Fig 4B and C, respectively) indicated that cannibalism led to the apoptosis of the engulfed cells (and occasionally of the cannibals), again similar to our observations in human tumours. Nevertheless, engulfed cells may be viable cells at first, because many of the inner cells observed displayed non-apoptotic morphology and some of them even appeared to undergo cell division (Fig 5A, arrow).

In order to determine whether Nupr1 inactivation was necessary for a PDAC cell to cannibalize or to be cannibalized, we generated separate populations of DsRed- and EGFP-expressing Panc-1 cells using lentiviral vectors. We knocked-down Nupr1 expression in EGFP-Panc-1 cells and mixed these cells with equal amounts of DsRed-Panc-1 cells transfected with control siRNA. As shown in Fig 4D, the majority of cannibal cells appeared among the green Nupr1-depleted population, whereas preys were either Nupr1-depleted or control cells. Thus, Nupr1-inactivation in the host cell appears mandatory for HoCC while the Nupr1 status of the prey does not appear as determinant criteria for engulfment. Hence, these experiments provide the first evidence that HoCC gives rise to cell-in-cells in PDAC, and of the ability of TGFβ to enhance it. Furthermore, they define Nupr1, a TGFβ-regulated chromatin regulatory protein, as a repressor of HoCC.

### Flow cytometry-based quantification of homotypic cell cannibalism

To render cell cannibalism amenable to quantification, we developed a fluorescence-activated cell sorting (FACS)-based method to measure cell-in-cell events, accountable for HoCC (Fig 5). We mixed equal numbers of DsRed- and EGFP-expressing Panc-1 cells prior to Nupr1-knockdown and treatment with TGFβ, and the resulting proportion of double-positive bodies were quantified (Fig 5A). We introduced a correction factor (*X*^2^) to our measurements, since green-in-green or red-in-red events, which are indistinguishable from single-positive individual cells in FACS, may occur with equal probability as red-in-green or green-in-red events. Results obtained from FACS measurements were comparable to direct counts of cell-in-cell events in microscopy fields of the same experiment, thus validating FACS-based quantification of cell cannibalism (Fig 5C). Both methods suggested that Nupr1-depletion favoured spontaneous cannibalism in Panc-1 cells (7.1 ± 0.8 *vs*. 4.4 ± 0.3% in siNupr1 and siCtrl, respectively, as measured by FACS). To establish an inverse relationship between Nupr1 expression and HoCC, we performed a similar FACS-based analysis of HoCC in several human PDAC cell lines (Capan-1, Capan-2, PATU8988T, PATU8902 and MiaPaCa2) with different levels of Nupr1 transcript as quantified by qRT-PCR (Fig 5D and Supporting Information Fig S2), which were rendered fluorescent by lentiviral transduction (MiaPaCa2) or by staining with fluorescent dyes CFSE and CMPTX before equinumeral mixing and 48 h culture. We found that the lowest the level of Nupr1 transcript (Capan-1), the more HoCC (44.88 ± 7.44%). Conversely, a similar approach revealed that Nupr1 overexpression in Panc-1 using lentiviral transduction inhibited HoCC compared to cells transduced with an empty vector (EV, Fig 5D, right). Finally, we found that gemcitabine, a common chemotherapy against PDAC which represses Nupr1 expression (Giroux et al, 2006), increased cell cannibalism in Panc-1 cells (Supporting Information Fig S3). Thus, we conclude that low Nupr1 expression elicits HoCC in PDAC cells.

### Impact of tumour microenvironnement settings on homotypic cell cannibalism

Empowered of a handy and objective FACS-based quantification of HoCC, we further quantified the impact of TGFβ in this phenomenon. Consistent with microscopy data, we observed that culture of control Panc-1 cells with TGFβ incremented HoCC and that, also in this setting, Nupr1-depleted cells were more inclined to HoCC than controls (10.2 ± 0.08 *vs*. 5.8 ± 0.9, Fig 5A and C). Since cannibalism has been proposed as a nutrient source upon starvation (Fais, 2007; Lugini et al, 2006; Wang et al, 2009), we assessed HoCC in control and Nupr1-depleted Panc-1 cells cultured in nutrient-free Earle's balanced salt solution (EBSS) medium (Fig 5E and Supporting Information Fig S4) and found no significant increase compared to mock. Similar results were obtained when Nupr1 expression was targeted by two additional siRNAs (Supporting Information Fig S5). Nevertheless, when the expression of the autophagy protein autophagy-related 5 homolog (ATG5) was inactivated using a specific siRNA, HoCC was significantly enhanced in control cells to levels comparable to Nupr1-depleted cells (12.67 ± 1.38% and 11.54 ± 0.48%, respectively). Thus, our data do not point to a major activation of HoCC upon starvation, unless the autophagy machinery is disrupted.

### HoCC and entosis are not driven by the same molecular pathways

Recently, a homotypic cell-in-cell phenomenon of entosis was described by which breast cancer cells could invade their siblings in an E-cadherin- and Rho-associated, coiled-coil containing protein kinase 1(ROCK1)-dependent manner (Overholtzer et al, 2007). To determine an eventual relationship between HoCC and entosis, we used our FACS-based HoCC quantification to analyze the impact of the inactivation of E-cadherin and ROCK1 on HoCC. Thus, we knocked-down the expression of these molecules in Panc-1-EGFP and Panc-1-DsRed cells using a specific siRNAs (Fig 5) and we assessed HoCC after 48 h, with or without concomitant Nupr1-depletion and/or TGFβ treatment. We found that, contrary to entosis, *CDH1* (encoding E-cadherin)-knockdown did not affect measurements of HoCC in either condition, whereas ROCK1 inactivation led to HoCC enhancement (Fig 5E). Concomitant inactivation of E-cadherin or ROCK1 did not significantly increase the effect of Nupr1-depletion. Therefore, HoCC in Nupr1-depleted PDAC cells do not share the molecular regulation by E-cadherin and ROCK1 with entosis.

### Nupr1 is not implicated in heterotypic cell cannibalism

Very convincing data documented the cannibalism of immune cells by melanoma cells (Lugini et al, 2006). We investigated whether PDAC cells were capable of engulfing living lymphocytes and the impact of Nupr1-depletion in this phenomenon. Thus, we knocked-down Nupr1 expression in Panc-1 cells 48 h prior to incubation with CFSE-stained peripheral blood mononuclear cells (PBMC) for 6 h, then we assessed the percentage of xeno-cannibalism by FACS as previously described (Lozupone et al, 2009; Lugini et al, 2006). Similar to melanoma, PDAC presented with xeno-cannibal activity towards living lymphocytes (60 ± 1.88% CFSE^+^ cells, Supporting Information Fig S6), although this was unaffected by Nupr1-depletion and TGFβ treatment. Thus, our data indicate that HoCC and heterotypic cell cannibalism do not share the requirement for Nupr1-inhibition.

### Inactivation of Nupr1 de-represses the transcription of phagocytosis-related genes

**Figure 6:**
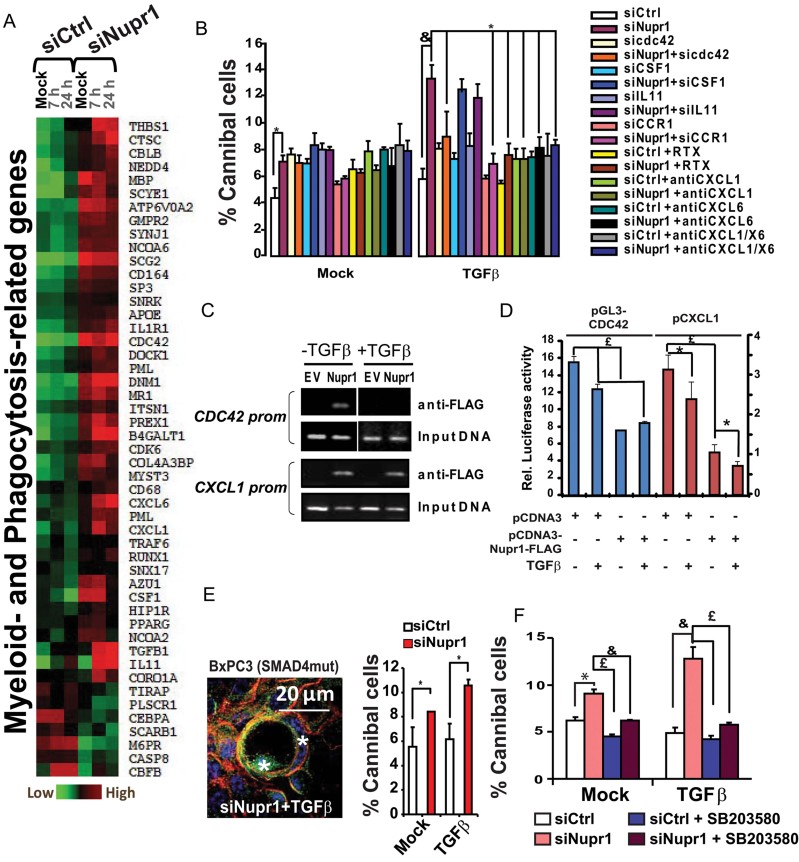
Nupr1-depletion favours expression of phagocytosis-related genes indispensable for HoCC in PDAC cells In the histograms, data are means ± SEM. *p ≤ 0.05, &*p* ≤ 0.01, £*p* < 0.005. Heatmap show myeloid- and phagocytosis-related (right) genes expression in control or Nupr1-depleted Panc-1 cells after mock- or TGFβ-treatment for 7 and 24 h.Histograms show the percentage of cannibal mock and TGFβ-treated Panc-1 cells that were previously transfected with the indicated siRNAs, and cultured with or without the CCR1/CCR2 antagonist repertaxin (RTX) or the neutralizing anti-CXCL1 and/or anti-CXCL6 antibodies, as indicated.Panc-1 cells were transfected with a Flag-tagged Nupr1 or an empty vector (EV) control, mock- or TGFβ-treated for 5 h, and chromatin immunoprecipitation (ChIP) was performed using an anti-Flag-tag antibody. The upper panels show occupancy of Nupr1 on *CDC42* and *CXCL1* promoters, the bottom panels for each promoter show DNA input (10%) before immunoprecipitation of EV and Nupr1-transfected lysates.*CDC42* and *CXCL1* gene reporter assay. Panc-1 cells were transfected with pGL3-CDC42 or pCXCL1 vectors along with prLucC2 vector and cultured with or without TGFβ. Histogram show normalized luciferase activity indicative of transcription activation.(Left) vinculin (green) and actin (red) immunostaining of Nupr1-depleted BxPC3 cells; (right) histogram shows FACS-based HoCC quantification of BxPC3 Nupr1-depleted and control cells upon TGFβ treatment. Nuclei were counterstained with DAPI.Histogram shows cytofluorometric quantification of HoCC in Nupr1-depleted and control cells that were treated with the indicated combination of TGFβ and the p38 inhibitor SB203580.

Given the molecular discrepancies we found between PDAC HoCC and other cell-in-cell phenomena described so far, we sought to characterize PDAC HoCC at the molecular level. Since we and others have shown Nupr1's involvement in the regulation of gene transcription and chromatin acetylation (Cano et al, 2011; Gironella et al, 2009; Kong et al, 2010; Sambasivan et al, 2009), we hypothesized that Nupr1-depletion would favour the expression of genes implicated in cell engulfment, and that TGFβ-induced gene profile would be affected. Therefore, we analyzed the transcriptome of Nupr1-depleted and control Panc-1 cells with and without TGFβ-stimulation (for 7 and 24 h) and screened for genes implicated in phagocytosis and phagocyte(myeloid) differentiation, as defined by the GO:0006909 and GO:0030099 lists from The Gene Ontology database, or using an unbiased GO-ANOVA analysis. Indeed, the results of this analysis revealed an upregulation of phagocytosis-related genes in Nupr1-depleted cells (Fig 6A, Supporting Information Fig S7 and Supporting Information Tables S2 and S3), and a deregulation of the expression of EMT-related genes (Supporting Information Fig S7 and Supporting Information Table S4). Consistent with the observations of cannibal cells in human pancreatic tumours, Nupr1-depleted Panc-1 cells displayed enhanced ectopic CD68 expression compared to controls. Thus, transcriptome data was congruent with our observations of cell cannibalism in human PDAC tissue and cells that, in the absence of Nupr1 and upon TGFβ treatment, PDAC cells become genetically programmed to cannibalize their siblings.

### Nupr1 inhibits HoCC by repressing the transcription of phagocytosis-related genes

We inferred that HoCC, like phagocytosis, is supported by chemotaxis and the concomitant cytoskeleton rearrangement of the cannibal and prey cells. Thus, we explored the contribution of a set of candidate genes selected among those upregulated in Nupr1-depleted Panc-1 cells that are implicated in phagocyte differentiation colony stimulating factor 1 (*CSF1*), chemotaxis (*IL11* (interleukin 11), *CXCL1* (chemokine (C-X-C motif) ligand 1) and *CXCL6* (chemokine (C-X-C motif) ligand 6)) and cytoskeleton remodelling cell division cycle 42 (*CDC42*). *CSF1*, *IL11* or *CDC42* were knocked-down in Nupr1-depleted Panc-1 cells using specific siRNAs, and CXCL1- and CXCL6 activities were blocked with neutralizing antibodies, and proportion of cannibal cells was assessed by FACS. As shown in Fig 6B and Supporting Information Fig S8, the inactivation of the cdc42 RhoGTPase and of CXCL1- and CXCL6-mediated chemotaxis abrogated TGFβ-induced HoCC in Nupr1-depleted cells. Similar results were obtained when CXCL1- and CXCL6-mediated chemotaxis were inhibited using a specific siRNA against their receptor (CCR1), or when the CCR1 antagonist, repertaxin (RTX), was introduced into the system. Thus, these results confirm that the repression of *CDC42*, *CXCL1* and *CXCL6* are critical milestones of Nupr1-mediated inhibition of HoCC.

To further establish the action of Nupr1 as a transcriptional regulator of cell cannibalism, we analyzed Nupr1 occupancy of the *CDC42* and *CXCL1* promoters by performing chromatin immunoprecipitation (ChIP) assays using Panc-1 cells transfected with a Flag-tagged Nupr1 or an EV. As shown in Fig 6C, Nupr1 binds to the *CDC42* and *CXCL1* promoters in untreated cells, demonstrating that they are indeed targets of Nupr1. Upon TGFβ-treatment, Nupr1 occupancy of the *CXCL1* promoter remained unchanged whereas *CDC42* promoter was freed from Nupr1, indicating that Nupr1 regulates these two promoters through different mechanisms. Luciferase-based gene reporter assays using the pGL3-CDC42 and the pCXCL1 reporters confirmed that overexpression of Nupr1 in Panc-1 cells leads to repression of the *CDC42* and *CXCL1* promoters, respectively (Fig 6D). Altogether, these results show that Nupr1 acts at the chromatin level on genes implicated in cell cannibalism.

### p38MAPK activity, but not Smad4, is necessary for HoCC in Nupr1-deficient PDAC cells

Considering that more than 50% of human PDAC present with inactivating mutations of the *Smad4/DPC4* gene, which is a key effector of TGFβ-signalling previously shown to participate in Nupr1 induction (Garcia-Montero et al, 2001), we determined whether Nupr1-mediated repression of HoCC upon TGFβ stimulation was affected by Smad4-inactivation. We generated EGFP- and DsRed-expressing batches of the Smad4-mutated BxPC3 pancreatic cancer cell line and determined whether such cells would perform cannibalism after Nupr1-depletion and TGFβ treatment. As shown in Fig 6E, Nupr1-depleted BxPC3 cells retained the ability to cannibalize upon TGFβ treatment at levels that were comparable to those observed in Panc-1 cells. Hence, this result indicates that the repressive function of Nupr1 over TGFβ-induced HoCC in PDAC cells is independent of Smad4. Since we previously showed that TGFβ may trigger Nupr1 expression in a Smad-independent manner through the p38MAPK (Malicet et al, 2003), we tested the effect of the p38MAPK inhibitor SB203580 in TGFβ-induced HoCC in Nupr1-depleted cells, and we found it reduced back to the basal levels observed in controls (Fig 6F and Supporting Information Fig S9). These results designate the p38MAPK activity as an essential actor of HoCC induction upon TGFβ treatment.

### Cell cannibalism is enhanced in *Nupr1*^*KO*^*;Pdx1-creER;LSL-KRAS*^*G12D*^*;INK4A/ARF*^*fl/fl*^ pancreatic tumours

**Figure 7:**
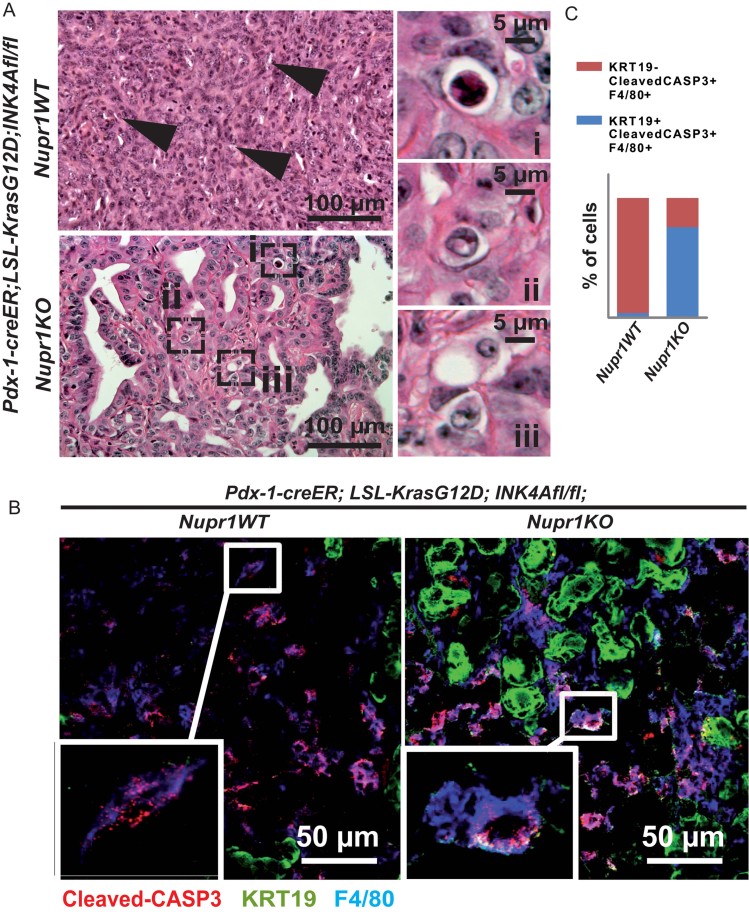
Nupr1-deficiency enables HoCC in murine pancreatic tumours Histological analysis of pancreatic tumours from *Nupr1WT versus Nupr1KO Pdx1-creER*; *LSL-Kras^G12D^INK4A/ARF^fl/fl^* mice. Pictures of H&E stained sections show numerous spindle-like cells (arrows) typical of poorly differentiated pancreatic adenocarcinoma in Nupr1WT tumours, and enrichment of cannibal cells (boxes i, ii and iii) in Nupr1KO tumours.Confocal microscopy pictures showing immunofluorescent staining of cleaved caspase-3 (red), the epithelial marker cytokeratin-19 (KRT19, green) and the phagocyte marker F4/80 (blue). Note that most cleaved-CASP3^+^ cells in Nupr1-deficient tumours display both KRT19 and F4/80.Histogram shows the fractions of KRT19^+^F4/80^+^ and KRT19^-^F4/80^+^ within the cleaved caspase-3 positive cells in tumour sections. Three specimens per genotype were analyzed and a minimum of 50 cells were counted per section.

Finally, in order to establish the pivotal role of Nupr1 in the repression of HoCC *in vivo*, we investigated whether Nupr1-deficiency resulted in more cell-in-cells in pancreatic tumours developed by the *Pdx1-creER*; *LSL-KRAS*^*G12D*^; *INK4A/ARF*^*fl/fl*^ mice (Bardeesy et al, 2006; Gu et al, 2002). To this end, we crossbred these mice with *Nupr1*^*KO*^ mice developed in our laboratory, and searched for cannibalism in the developed pancreatic tumours. As predicted, and similar to our results in human tumours, histological analysis of *Nupr1*^*KO*^ pancreatic tumours (*n* = 3) revealed a higher frequency of cell-in-cell figures (Fig 7A) compared to their rare occurrence in tumours arising in the *Nupr1*^*WT*^ background (*n* = 6; frequency 2.84 ± 0.6% *vs*. 0.49 ± 0.7%, respectively; *p* = 0.038). Moreover, a fraction of *Nupr1*^*KO*^ PDAC cells, mainly located in the periphery of glandular patches, co-expressed the epithelial marker KRT19 and the phagocyte marker F4/80 (Fig 7B and Supporting Information Fig S10), consistent with *in vitro* data showing ectopic expression of phagocyte-related genes in the absence of Nupr1. Such mixed epithelial/phagocytic phenotype was rarely detected in Nupr1^WT^; KRAS^G12D^; INK4A/ARF^null^ tumours. More importantly, and again in agreement with *in vitro* data, cleaved caspase-3 staining showed that the majority of KRT19^+^F4/80^+^ cells are apoptotic within Nupr1^KO^; KRAS^G12D^; INK4A/ARF^null^ tumours, in stark contrast with their Nupr1^WT^ counterparts, in which most apoptotic cells were found to be KRT19^-^F4/80^+^ macrophages (Fig 7B and C). These observations support our hypothesis that, in the absence of Nupr1, pancreatic epithelial cancer cells aberrantly acquire phagocyte-like properties, including HoCC, which ultimately lead to cell death. Unfortunately, metastasis formation could not be studied on this model because mice developed tumours very rapidly and died.

## DISCUSSION

In this article, we present formal evidence of the ability of PDAC cells to perform HoCC both *in vivo* and *in vitro*, thereby forming the cell-in-cells figures that are observed in human pancreatic tumours. After establishing an inverse correlation between this phenomenon and metastasis appearance in human PDAC patients, we show that PDAC HoCC leads to cell death which offers a possible explanation for metastasis inhibition. At the molecular level, we show that HoCC is allowed by Nupr1-deficiency, and that TGFβ is able to enhance this cellular process. By a large-scale transcriptome analysis of Nupr1-depleted PDAC cells, we evidenced that HoCC is associated to a shift of the genetic program in these cells towards a pro-phagocytoid profile. Moreover, the inactivation of phagocytosis-related genes that are overexpressed in the absence of Nupr1 impairs the cannibal response induced by TGFβ in Nupr1-depleted cells, thus, establishing that Nupr1 represses HoCC in PDAC cells by repressing the expression of key genes implicated in this process. Finally, the genetic inactivation of Nupr1-enhanced cell cannibalism in Kras^G12D^ INK4a^null^ murine pancreatic tumours, indicating that Nupr1 expression antagonizes cell cannibalism *in vivo* within the primary tumour.

Cell-in-cells have been reported in human malignancies for many years (see Fais, 2007, and Overholtzer & Brugge, 2008, for review), but the interest for a tumour cell to form such structures has remained a matter of debate. Finding a definite and unique answer to this question can be complex, since several different processes have been reported so far to give rise to cell-in-cells, including emperipolesis (Burns, 1967; Humble et al, 1956; Shamoto, 1981), heterotypic cell cannibalism (Lozupone et al, 2009; Lugini et al, 2006; Wang et al, 2009), homotypic cell-in-cells and/or entosis (Abodief et al, 2006; Gupta & Dey, 2003; Kojima et al, 1998; Kumar et al, 2001; Overholtzer et al, 2007), and the additional type reported here of Nupr1-repressed HoCC in pancreatic cancer cells. In contrast to our conclusions about an inverse relationship between HoCC and metastasis in PDAC, a number of studies have suggested a tumour promoting role for cell cannibalism. Among them, it was convincingly documented that melanoma cells are able to cannibalize immune cells and to feed on them, thereby ensuring their survival upon starvation and probably leading to immune escape (Lugini et al, 2006). We found that, while PDAC cells are able to cannibalize living lymphocytes, this heterotypic or xeno-cannibalism is unaffected by Nupr1-inactivation and TGFβ treatment, hence, it is not related to HoCC at the molecular level in PDAC. Moreover, we found that starvation did not enhance HoCC of PDAC cells significantly, in agreement with previous data on HoCC of small cell carcinoma of the lung (SCCL) (Brouwer et al, 1984), and indicating that HoCC is not a major survival response to metabolic stress in PDAC, as proposed for other cancers (Malorni et al, 2007; Matarrese et al, 2008). Consistently, we found that, on the contrary to self-cannibalism (autophagy) and entosis (Florey et al, 2011), HoCC in PDAC cells did not require the ATG5, and that its inactivation would rather stimulate HoCC, arguing for a function of HoCC in PDAC different from the above mentioned cell-in-cell processes. Finally, we believe that the comprehensive histological analyses performed on effusions, crush smears and fluids (urine) from medulloblastoma, urothelial and breast cancer patients, which revealed a correlation between the presence of homotypic cell-in-cells and tumour progression (Gupta & Dey, 2003; Kojima et al, 1998; Kumar et al, 2001) could account for entosis or emperipolesis, which are more likely to operate in detached cancer cells. Further analysis of Nupr1 expression and cell death in these types of samples could be helpful solving this question. Our data presented here suggest that in PDAC, the homotypic engulfment of a tumour cell by another tumour cell is a putative suicidal mechanism that is strongly repressed by the high expression of Nupr1 in PDAC (Su et al, 2001a, b). Another important point is that we found a positive effect of TGFβ on HoCC, which is in agreement with a previous report from Brouwer et al (1984), indicating that HoCC is triggered in SCCL by a soluble serum-factor unknown at that time. Together, our data and Brouwer's give evidence that HoCC is deleterious for cancer cells, is not triggered by starvation but is dependent on a serum factor, which we identified to be the TGFβ.

Studies from Overholtzer et al (2007) reported that entosis in breast cancer cells is dependent on a ROCK-mediated Rho-GTPase activity triggered after E-cadherin-mediated cell-cell contact. Our experiments ruled out an eventual identity between HoCC and entosis, since the former is not inhibited by loss of ROCK1 (Fig 5E), but is instead fueled by the activity of the cdc42 GTPase (Fig 6). In addition, E-cadherin inactivation did not affect HoCC, which was also to expect, since TGFβ induces E-cadherin down-regulation while enhancing HoCC. Although, we cannot exclude that HoCC may rely on cell-cell contacts mediated by other types of cadherins such as N-cadherin, whose expression is induced by TGFβ.

Epithelial-to-mesenchymal transition is at the basis of the invasive capacity of PDAC cells. Here, we show that TGFβ, which is the main inductor of EMT in PDAC, has also the potential to enhance HoCC, given that Nupr1 is inactivated. On the same line, we have recently demonstrated that Nupr1 mediates in PDAC cells the key milestones of metastasis that are TGFβ-induced migration, invasiveness and regulation of cell adhesion, through repression of *CDC42* expression (Sandi et al, 2011). Given the data presented in this manuscript and our prior reports, we propose that Nupr1 may be a key switch of cellular physiological plasticity that favours an aggressive, stress-resistant and metastasis-prone phenotype through EMT, and represses a cell deleterious cannibalism-prone phenotype. In support to a pro-metastatic role of Nupr1, Ree et al (1999) have reported that Nupr1 overexpression is pivotal to the breast cancer cells that develop metastasis in the central nervous system.

In our hypothetical model, given a genetic context where Nupr1 expression is intact and potentiated by TGFβ, PDAC cells undergo EMT and become able to detach from the malignant glandular lesions, to migrate and to invade secondary tissues. On the other hand, when a genetic or an epigenetic event inactivates Nupr1 expression, a loosely anchored PDAC cell stimulated by TGFβ would be inclined to HoCC-associated cell death. Indeed, stimulation by TGFβ results in a 20–40% enhancement of HoCC in Nupr1-depleted cells (Fig 5) and in a similar effect on the overexpression of phagocyte-related genes such as *CD68*, *CDC42* and *CXCL1* in these cells (Fig 6). Of note, TGFβ may also be implicated in enhancement of ‘basal’ HoCC in Nupr1-depleted cells, since TGFβ1 is overexpressed in these cells (Supporting Information Fig S3 and Supporting Information Table S4). Nonetheless, we do not exclude that tumour microenvironmental factors or stresses other than TGFβ can induce HoCC *in vivo*.

A possible association between HoCC and good prognosis of patients with cancer was previously suggested from the observation in human giant cell (OCGT) pancreatic carcinomas, which present with a better prognosis compared to anaplastic PDAC, reaching post-operatory survival of over 10 years in some cases (Deckard-Janatpour et al, 1998). OCGT pancreatic carcinomas are rare (less than 1% of pancreatic cancer cases), discouraging in depth research about the mechanisms underlying cell cannibalism. Our observation here that around 30% of PDAC analyzed presented HoCC, and that patients in which this process is detected develop less metastasis, encourages to believe that pharmaceutical modulation of this process may result in an effective treatment for this deadly disease. Therefore, a better understanding of the molecular pathways implicated in HoCC may yield new therapeutic targets for PDAC treatment. We report here the first known soluble HoCC-inducing factor, TGFβ, and delineated a TGFβ-regulated pathway that favours HoCC in PDAC cells. Depending on the inhibition of Nupr1 expression, as well as TGFβ-induced activation of the p38MAPK pathway and concomitant transcription of the *CDC42*, *CXCL1* and *CXCL6* genes, cells become able to cannibalize other cells. Therefore, Nupr1 appears as a seducing target for the development of a therapeutic strategy against PDAC. Moreover, our finding that p38MAPK activity, but not Smad4, is required for HoCC indicates that targeting of this phenomenon may be applied in all pancreatic cancers and not only in the barely half of patient with unmutated *Smad4/DPC4* gene.

## MATERIALS AND METHODS

### Patients and histology

Pancreatic adenocarcinoma specimens analyzed in this study were collected at surgery from 36 patients with available clinical history, at the Gastroenterology Department of the Hôpital Nord de Marseille, France. Pancreas tumour specimens from patients and Nupr1^KO^ or ^WT^; Kras^G12D^; Ink4a^null^ mice were formalin-fixed and paraffin-embedded. Sections were immunostained in a BENCHMARK XT automate from Ventana-Roche. Hematoxilin/eosin (Vector Laboratories) staining was performed following standard protocols. Five to ten sections covering the overall dissected tumour mass were analyzed for the presence of cannibal cells. For calculation of HoCC frequency (percentage of cannibal cells per sample), cells from three 20× magnification fields were counted per sample, and cannibalism was expressed as the mean ± SEM of three different samples per cohort. No less than 150 cells were counted per field. For frozen sections of murine tumours, tissues were immediately immerged in ice cold methyl butane (Sigma) and frozen on liquid nitrogen. Frozen tissues were embedded in OCT (Sakura Finetek) before slicing into 10 µm sections on a Leica Cryostat. Mounting was performed using Eukitt solution (Vector). Staining was analyzed with an Olympus BX61 automated microscope. All tissues were collected via standardized operative procedures approved by the Institutional Ethical Board and in accordance with the Declaration of Helsinki. Informed consent was obtained for all tissue samples linked with clinical data.

### Cell culture and reagents

Human Panc-1, Capan-1, Capan-2, MiaPaCa2 and BxPC3 (pancreatic cancer) and 293-T (embryonic kidney) cell lines were from ATCC. PATU8988T and PATU8902 were from the DSMZ (Braunschweig). Panc-1, MiaPaCa2, 293-T, PATU8988T and PATU8902 were cultured in Glutamax-containing DMEM (Invitrogen), and BxPC3, Capan-1 and Capan-2 in RPMI (Invitrogen), supplemented with 10% FBS (PAA Laboratories), at 37°C, 5% CO_2_, H_2_O saturated. Nutrient deprivation was performed in EBSS medium (Life Technologies). TGFβ1 (Peprotech) was used at 10 ng/ml and CXCL1/CXCL6 antagonist RTX (Sigma) at 250 nM. Neutralizing anti-CXCL1 and anti-CXCL6 antibodies (R&D) were used at 5 ng/ml and 40 ng/ml, respectively.

### Animals

Mice bearing a homozygous deletion of exon 2 of the *Nupr1* gene were reported previously (Vasseur et al, 2003). These mice are viable and fertile and exhibit normal pancreas development (Vasseur et al, 2004). *Pdx1-creER*; *LSL-Kras*^*G12D*^; *INK4A/ARF*^*fl/fl*^ mice were obtained by crossbreeding of the following strains: *Pdx1-CreER* (Gu et al, 2002) (kindly given by D. Melton), *LSL-Kras*^*G12D*^ (Aguirre et al, 2003) and *INK4A/ARF*^*fl/fl*^ (Aguirre et al, 2003; Bardeesy et al, 2006) and (kindly given by R. Depinho, Dana-Faber Cancer Institute, MA). At birth, mothers were isolated with their progeny and fed *ad libitum* with soy-free tamoxifen (400 mg/kg)-containing chow (Teklad CRD TAM400/CreER, Harlan, Teklad (Kiermayer et al, 2007) until weaning. Care and manipulation of mice were performed in accordance with national and European legislation on animal experimentation and were approved by the Aix-Marseille University Institutional Animal Care and Use Committee.

### Antibodies

Primary monoclonal antibodies were developed in mouse, excepting the anti-Nupr1 and the Alexa fluor 647-conjugated anti-mouse F4/80 antibodies (Serotec), which were rat monoclonals. Antibodies against vimentin, E-cadherin, vinculin, β-tubulin and β-actin were from Sigma. Anti-αSMA and N-cadherin were from Abcam. The M30 CytoDEATH^©^ antibody was from Alexis Biochemicals and the mouse monoclonal anti-GFP from Roche. For immunohistofluorescence, the following polyclonal antibodies were used: anti-KRT19 (Santa Cruz) and rabbit anti-cleaved-caspase 3 (clone Asp175, Cell Signalling). Monoclonal antibodies against EMA, AE1E3, β-catenin and CD68 (PG-M1 clone) used in immunohistochemistry experiments were from Dako. Alexa Fluor 647-conjugated phalloidin and mouse specific secondary antibodies conjugated to Alexa Fluor 488 and 546 were from Molecular Probes.

### siRNA-mediated gene silencing

Cells were seeded in 6-wells plates to reach 30–50% confluence. Twenty-four hours later, a mix containing 2.2 pmol. of siRNA and 8 µl of the transfection reagent INTERFERIN (Polyplus Transfection) was added to the culture medium according to manufacturer's instructions. Cells were further incubated for 24 or 48 h before TGFβ1 treatment without change of medium. Control, *Nupr1-* (siNupr1-4, siNupr1-1 and siNupr1-2) and *ATG5*-specific siRNAs were described elsewhere (Giroux et al, 2006). siNupr1-4 was used in all experiments, unless those of Supporting Information Fig S5. Predesigned siRNAs against *ROCK1* (Hs_ROCK1_9), *CDC42* (Hs_CDC42_7), *CSF1* (Hs_CSF1_2), *IL11* (Hs_IL11_4) and *CCR1* (Hs_CCR1_1) were from Qiagen. *CDH1*-specific siRNA (NM_004360si.1) was from Eurofins MWG.

### Generation of EGFP- and DsRed-expressing pancreatic cancer cell lines

Fluorescent Panc-1, MiaPaCa-2 and BxPC3 cells were obtained by transduction of lentiviral particles generated using the pCCL-EGFP (kindly provided by C. Raoul) and pLVX-DsRed-N1 (Clontech) plasmids, respectively. Vesicular stomatitis virus G (VSV-G)-pseudotyped lentiviral particles were obtained by co-transfection of the above mentioned plasmids along with the packaging and envelope-encoding plasmids 2 into 293-T cells, using Lipofectamine 2000 (Invitrogen) and according to manufacturer's instructions. Lentivirus-containing supernatants were collected after 24 and 48 h, and then added to Panc-1 cells cultures at 0.5× concentration in fresh medium. Two sequential rounds of transduction were performed at 24 h interval. Seventy-two hours after transduction, more than 90% of the cells displayed fluorescence (data not shown). DsRed-Panc-1 cells were further selected by culture in presence of 2 µg/ml puromycin.

### PDAC cell staining with CFSE and CMPTX

Cells were washed in PBS and incubated with 5 mM CFSE or 5 µg/ml CMPTX (Molecular Probes) in OPTIMEM medium (Life Technologies), at 37°C, 5% CO_2_, for 20 min. Then cells were thoroughly washed with PBS and cultured for a supplementary hour at 37°C before mixing with PBMC, or overnight for mixing between CFSE- and CMPTX-stained cells.

### Flow cytometry-based quantification of HoCC

For cell cannibalism quantification, stable EGFP- and DsRed- cells, and transiently CFSE- or CMPTX-labelled cells, were mixed at 1:1 ratio prior to or after gene silencing, as indicated, and cultured with or without TGFβ1 for 48 h. Then, cells were trypsinized and analyzed immediately in a FACSCalibur (Becton Dickinson). At least five thousand events of each sample were acquired in duplicates or triplicates. ‘Live cells’ gate was delineated after debris exclusion using FSC/SSC dot plots and doublets were excluded using channel-area/channel-width dot plots. Enhanced size and granularity (increased ∼2-fold) of the double-positive population compared to the single-positive populations were consistent with enlarged cell-in-cell bodies with perturbed intracellular structure (Fig 5B). Data were analyzed using the FlowJo 7.5 software (Tree Star, Inc.).

### Xeno-cannibalism assay

Assessment of heterotypic cannibalism of living blood cells by PDAC cells was performed after (Lugini et al, 2006). Briefly, PBMC were obtained from fresh buffy coats, stained with CFSE as described, and cultured for an additional hour before thorough washing in cold medium, counting and mixing with Panc-1 cells. Panc-1 cells were transfected with siCtrl or siNupr1-4 as described above. Forty-eight hours later, siRNA-transfected Panc-1 cells were mixed with CFSE-stained PBMC at 1:10 ratio and incubated at 37°C, CO_2_, or at 4°C for control, during 6 h. Ten thousand events were acquired on a FACSCalibur and analyzed using FlowJo. Panc-1 cells showing green fluorescence were considered xeno-cannibal.

### Immunofluorescence

Cells were seeded in glass cover slips prior to gene silencing as described above, then cultured with or without TGFβ1 for the times indicated. Immunofluorescent stainings on cells and tissues were performed essentially as previously described (Gommeaux et al, 2007; Taieb et al, 2008). Slides were examined on a Nikon microscope Eclipse 90I. Z-stack pictures were obtained using a Nikon Digital Sight DS-1QM camera controlled by the NIS element AR software (Nikon). For confocal microscopy, pictures were assessed in a Zeiss 510 Meta microscope and analyzed using the Zeiss LSM Browser 4.0.0.157 software, at the PICsL (Plateforme d'Imagerie Commune du Site de Luminy) facilities.

### Chromatin immunoprecipitation (ChIP) assay

ChIP assay was performed using the EZ-ChIP kit (Millipore) according to the manufacturer's instructions. Input DNA was collected after pre-clearing lysate with Protein G/agarose beads prior to immunoprecipitation with the anti-FLAG (M2) antibody (Sigma). PCR was performed using TaKaRa LA Taq with GC Buffer II, according to manufacturer's suggestions (Takara Bio Inc). A 230-bp region of the CDC42 promoter was amplified by PCR, using specific primers (5′-GCCAGTGATCCCAGCTACTC-3′, 5′-GGGCTATGCTCTGCATGTTT-3′), and a 260-bp region of the CXCL1 promoter was amplified by PCR, using specific primers (5′-CCCTGGTGTCATGTGCTATG-3′, 5′-CCAGTGTTAGCGTCAGTGGA-3′).

**The paper explained**

PROBLEM:

Cell-in-cell structures can arise in PDAC from unknown origins and mechanisms. We investigated whether cell cannibalism gives rise to these structures, identified the molecules involved, and explore the roles of the chromatin factor Nupr1. Moreover, we determined the frequency of these events in a cohort of 36 human PDAC samples and searched for an eventual relationship between cell cannibalism and patients' prognosis.

RESULTS:

Thirteen out of 36 human PDAC samples analyzed presented with cells-in-cells, which displayed epithelial and phagocyte markers but did not express Nupr1. Patients with cell-in-cell-containing tumours were found to develop significantly less metastasis than those without. Nupr1-knockdown in PDAC cell lines led to outlines of cell-in-cells that formed from HoCC; the frequency of which was significantly increased following stimulation of cells with TGFβ, with cells ultimately dying. Transcriptional analysis of Nupr1-depleted cells revealed that HoCC was accompanied by upregulation of phagocytosis-related genes. Among these genes, inactivation of *CDC42*, *CXCL1*, and *CXCL6* prevented TGFβ-induced cannibalism in Nupr1-depleted cells. *Nupr1*^*KO*^ *Kras*^*G12D*^ *INK4a*^*null*^ pancreatic cancer cells had increased levels of HoCC compared to their Nupr1^*WT*^ counterparts.

IMPACT:

Homotypic cell cannibalism was found to be inversely correlated to metastasis, in contrast to other types of cell cannibalism previously described. This process was found to be repressed by Nupr1, which is also responsible for repression of phagocytosis-related TGFβ-induced target genes that are required for HoCC. Hence, it appears from these findings that modulation of HoCC by genetic or chemical manipulation of he TGFβ- > Nupr1 axis may constitute a potential therapeutic strategy for treatment of pancreatic cancer.

### Luciferase gene reporter assays

pGL3-CDC42 (WT) (Liu et al, 2011) and pCXCL1 (Botton et al, 2011) vectors were kindly given by Dr Yi Zheng (Children's Hospital Research Foundation, Cincinnati, USA) and Dr Stephane Rocchi (C3M, INSERM U895, Nice, France), respectively. Panc-1 cells were transfected with the reporter vectors, prLucC2-Renilla vector and empty pCDNA3 or pCDNA3-Nupr1-FLAG using Lipofectamin 2000 (Invitrogen), according to manufacturer's instructions. Twenty-four h later, cells were mock- or TGFβ-treated for 6 h before cell lysis using the 5× cell lysis buffer (Promega). Firefly and Renilla luciferase activity were assessed using the Luciferase® Reporter Assay (Promega) following the manufacturer's instructions. Results are expressed in arbitrary unit of relative firefly activity normalized by Renilla activity.

### Western blotting

Forty-eight hours after gene silencing, cells were incubated for 24 hrs with TGFβ1 then, lysed in lysis buffer (Taieb et al, 2008) and centrifuged at 13,000 rpm in a table centrifuge for 10 min at 4°C. Sixty micrograms of protein lysates were separated in a 12% SDS–PAGE, transferred onto nitrocellulose filters and revealed following standard protocols and using a Fusion X7 Imager (Bilber Lourmat).

### RNA extraction and quantitative RT-PCR

Total RNA from cells was extracted using TRIzol (Invitrogen) and cDNAs were prepared using ImProm-IITM kit (Promega, Madison, WI) following manufacturer's instructions. Quantitative PCR was performed in a LightCycler® (Roche, Basel, Switzerland), using the SYBR® Premix Ex TaqTM (Takara Bio, Madison, WI). Amplification consisted in an initial 10 s denaturation at 95°C followed by cycles of 8 s denaturation at 95°C, 6–12 s annealing at 55–57°C (according to primers) and 12–25 s of extension at 72°C. Melting curve was obtained by heating at 20°C/s to 95°C, cooling at 20°C/s to 65°C and heating at 0.1°C/s to 95°C with fluorescence data collection at 0.1°C intervals. Quantization was performed using the RealQuant software (Roche). Primers sequences are available upon request.

### DNA microarray

Fifteen micrograms of total RNA were reverse transcribed into cDNA using Superscripts reverse transcriptase (Invitrogen) and T7-oligo-d(T)24 (Geneset) as a primer. Second-strand synthesis was performed using T4 DNA polymerase and *E. coli* DNA ligase, and then phosphorylated blunt ends were generated using T4 polynucleotide kinase. cDNA was purified by phenol–chloroform extraction using phase lock gels (Brinkmann). Then, cDNAs were *in vitro* transcribed for 16 h at 37°C by using the IVT Labelling Kit (Affymetrix) to produce biotinylated cRNAs. Labelled cRNA was purified using the RNeasy Mini Kit column (QIAGEN). Purified cRNA was fragmented to 200–300 mers using a fragmentation buffer. The quality of total RNA, cDNA synthesis, cRNA amplification and fragmentation was monitored by capillary electrophoresis (Bioanalyzer 2100, Agilent Technologies). Fifteen micrograms of fragmented cRNA were hybridized for 16 h at 45°C under constant rotation, using a human oligonucleotide array U133 Plus 2.0 (Genechip, Affymetrix). After hybridization, chips were processed using the Affymetrix GeneChip Fluidic Station 450 (protocol EukGE-WS2v5_450). Staining was made with streptavidin-conjugated phycoerythrin (SAPE, Molecular Probes), followed by amplification with a biotinylated anti-streptavidin antibody (Vector Laboratories), and followed by a second round of SAPE. Chips were scanned using a GeneChip Scanner 3000 G7 (Affymetrix) enabled for high-resolution scanning. Images were extracted with the GeneChip Operating Software (Affymetrix GCOS v1.4). Quality control of microarray chips was performed using the AffyQCReport software (Gautier et al, 2004). A comparable quality between microarrays was demanded for all microarrays within each experiment. Microarray data are available from Gene Expression Omnibus (National Center for Biotechnology Information, USA) and ArrayExpress (European Bioinformatics Institute, UK) under the accession numbers GSE38772 and E-MTAB-1176, respectively.

### Statistics

Statistical analyses were performed using the Student's *t*-test or Mann–Whitney test for small samples. Values were expressed as mean ± SEM, with significance set at *p* ≤ 0.05. qRT-PCR and FACS data are representative of at least three independent experiments in each case. For microarray analysis, background subtraction and normalization of probe set intensities were performed using the method of robust multiarray analysis (RMA) described by Irizarry et al (2003). To identify differentially expressed genes, gene expression intensity was compared using a moderated *t*-test and a Bayes smoothing approach developed for a low number of replicates (Smyth, 2004). To correct for the effect of multiple testing, the false discovery rate, was estimated from p values derived from the moderated *t*-test statistics (Benjamini et al, 2001). The analysis was performed using the affylmGUI Graphical User Interface for the limma microarray package (Wettenhall et al, 2006).

## Supplementary material

Detailed facts of importance to specialist readers are published as ”Supporting Information”. Such documents are peer-reviewed, but not copy-edited or typeset. They are made available as submitted by the authors.
