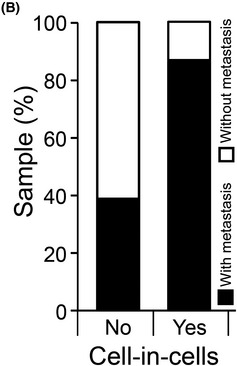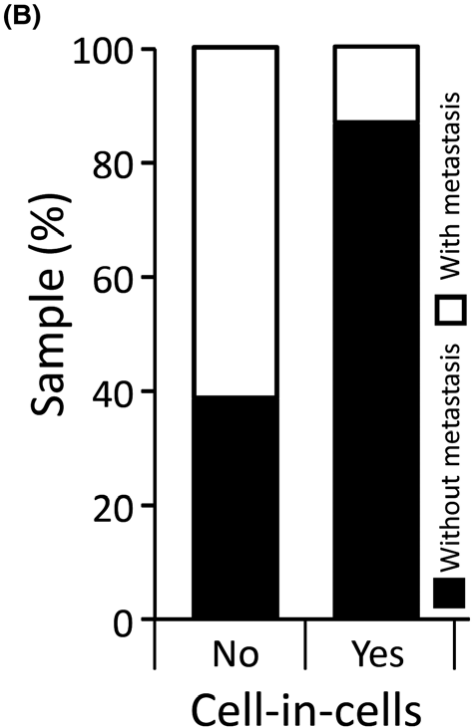# Homotypic cell cannibalism, a cell‐death process regulated by the nuclear protein 1, opposes to metastasis in pancreatic cancer

**DOI:** 10.15252/emmm.202114243

**Published:** 2021-05-07

**Authors:** Carla E Cano, María José Sandí, Tewfik Hamidi, Ezequiel L Calvo, Olivier Turrini, Laurent Bartholin, Céline Loncle, Véronique Secq, Stéphane Garcia, Gwen Lomberk, Guido Kroemer, Raul Urrutia, Juan L Iovanna

## Abstract

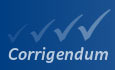

The authors became aware that the labeling in Figure 1B had been inverted. Source data for this figure were correctly labeled and published in the original paper as Supplementary Table 1. The authors apologize for the mistake and any confusion it may have caused.