# Supplementary material for: Homotypic cell cannibalism, a cell-death process regulated by the nuclear protein 1, opposes to metastasis in pancreatic cancer
Source: EMBO Mol Med. 2012 Jul 23;4(9):964–79. doi: 10.1002/emmm.201201255 (PMC3491828; doi:10.1002/emmm.201201255)
Supplement: Supplementary file 1 [file emmm0004-0964-SD1.pdf]

EMBO
Molecular Medicine

Manuscript EMM-2012-01255

Homotypic cell cannibalism, a cell-death process regulated by the Nuclear Protein 1, opposes to metastasis in pancreatic cancer

Carla E Cano, María José Sandí, Tewfik Hamidi, Ezequiel L Calvo, Olivier Turrini, Laurent Bartholin, Céline Loncle, Véronique Secq, Stéphane Garcia, Gwen Lomberk, Guido Kroemer, Raul Urrutia, and Juan L Iovanna

Corresponding author: Juan Iovanna, INSERM U1068

Review timeline:

Submission date:	03 February 2012
Editorial Decision:	02 March 2012
Revision received:	11 May 2012
Editorial Decision:	13 June 2012
Revision received:	15 June 2012
Accepted:	20 June 2012

Transaction Report:

(Note: With the exception of the correction of typographical or spelling errors that could be a source of ambiguity, letters and reports are not edited. The original formatting of letters and referee reports may not be reflected in this compilation.)

1st Editorial Decision 02 March 2012

Thank you for the submission of your manuscript to EMBO Molecular Medicine. We have now heard back from the two out of three referees whom we asked to evaluate your manuscript. As the third reviewer is quite late, and the other two reports are consistent, we thought that making a decision at this stage would be appropriate. I will forward to you the last review when it will become available.

As you will see from the enclosed reports, both referees find the study interesting and well done. Nevertheless, they both raise some issues that need addressing in a major revision of your manuscript.

In particular, referee #1 would like some clarifications regarding the terminology of the described phenomenon: entosis vs. cell cannibalism. In addition, this referee mentions additional studies that should be cited and discussed, as they are directly relevant.

In terms of extra-experiments, both referees make interesting suggestions to strengthen the conclusions and substantiate the claims that I believe would be important to do.

Given the balance of these evaluations, we feel that we can consider a revision of your manuscript if you can address the issues that have been raised within the space and time constraints outlined below. Please note that it is EMBO Molecular Medicine policy to allow only a single round of revision and that, as acceptance or rejection of the manuscript will depend on another round of review, your responses should be as complete as possible.

Revised manuscripts should be submitted within three months of a request for revision; they will otherwise be treated as new submissions, except under exceptional circumstances in which a short extension is obtained from the editor. Also, the length of the revised manuscript may not exceed 60,000 characters (including spaces) and, including figures, the paper must ultimately fit onto optimally ten pages of the journal. You may consider including any peripheral data (but not methods in their entirety) in the form of Supplementary information.

I look forward to seeing a revised form of your manuscript as soon as possible.

Yours sincerely,

Editor
EMBO Molecular Medicine

***** Reviewer's comments *****

Referee #1:

In this study Cana et al. performed an extensive series of cellular, biochemical, and molecular analyses on cell cannibalism in cancer. In particular, they carefully analyze cell cannibalism in pancreatic adenocarcinoma. Both histopathological analyses and in vitro studies have been carried out. The authors found pictures of cell cannibalism (also called "cell in cell") in samples from patients with pancreatic cancer. The authors also found that patients with pancreatic adenocarcinoma whose tumors display cell cannibalism develop less metastasis than those without. Several in vitro analyses have been performed in order to investigate the mechanisms underlying cell cannibalism. At least in vitro, cell cannibalism was found enhanced by TGFbeta and repressed by the Nuclear protein 1 (Nupr1) in pancreatic cancer cells. The authors conclude suggesting that inactivation of Nupr1 provokes a genetic reprogramming in PDAC cells that elicit cell cannibalism-associated cell-death in vitro and in vivo and that this behavior could exert a metastasis suppressor activity. Finally, the authors also suggest the potential implication of cell cannibalism (its induction) as a target in anticancer therapy.

I found this work very interesting and clearly written. It contains a number of very interesting findings, including some possible mechanism leading to cell cannibalism in pancreatic cancer cells. However, I have some points that must be considered and discussed in detail.

Major points

The major point is a general and conceptual issue. The idea that cell-in-cell (i.e. entosis) could be considered here is puzzling. The idea of these authors, if I have well understood, is that a small percentage of cancer cells are capable of engulfing and cannibalize their siblings. The authors seem to consider cell cannibalism as an "active" process of phagocytosis by non-professional phagocytes. This means that this is not entosis. Cell cannibalism was described several years ago by different authors (e.g. Steinhaus J. Virchows Arch 1891; Stroebe H. Beitrage Pathol 1892; and more recently: DeSimone et al 1980), but several further works have been carried out on this matter in different models (e.g. Mormone et al. 2006; Matarrese et al. 2011; Lai et al. 2010). A more careful analysis of literature must be included in this work).
Authors please comment carefully. It is very important to discriminate between entosis or cannibalism.

Although clinical significance of cell cannibalism in cancer still remains unclear, there is a plethora of works that should be considered (cited) and discussed in this manuscript (among these Kojima et al. Acta Cytol 1998; Kumar et al. Acta Cytol 2001; Matyarrese et al 2008; Gupta et al 2003; Malorni et al. 2007; Abodief et al. 2006). These works suggest that, at variance with the hypothesis raised by these authors, cell cannibalism could represent: i) a cell survival mechanism for host cell and ii) a "negative" prognostic factor. This sounds very trustworthy.
Authors please comment. It could be hypothesized that pancreatic cancer could differ from other forms of cancer and/or that gemcitabine treatment could exert an inhibitory effect on cell cannibalism. Please check (in vitro).

The authors found "a strong relationship between cannibalism and metastasis suggesting an anti-metastasis role of cell-in-cell structures". I am not convinced by the power of this analysis based on very few patients. More prudent statements should be provided.

In a previous work Cana and coauthors (2011) hypothesize that Nupr1 could modulate autophagy. The authors should evaluate some autophagic marker in their study. In fact, some papers in literature hypothesize a cross-talk between autophagy and cannibalism. I saw in literature that under starvation cell cannibalism might increase (more than inactivation of Nupr1 can do). Please check.

A further important point concerns the ability to engulf other cells. The authors say: "engulfed cells may be viable cells". Literature on the argument is very poor. However, as suggested by Lugini et al (2006) metastatic melanoma cells are capable of engulfing and digest live autologous CD8 lymphocytes. It could be of great interest to know if pancreatic cells do the same. On the basis of the results reported by the authors, I expect that these cells could not be able to cannibalize live lymphocytes that should kill them. This could at least partially explain the disparity with literature (in terms of prognosis).

A further important point concerns cytoskeleton involvement in the cell cannibalism. The proposed model (Nupr1-inactivation, TGFbeta effects) seems to represent a useful model to investigate host cell cytoskeleton. In particular, it could be very interesting to evaluate Rho family small GTPase in the system. In fact, at least two papers (Fiorentini et al 2001; Overholtzer et al. 2007) hypothesize a role for these proteins in cytoskeleton dynamics and cell engulfment. Since activation of Rho signals via TGF- -induced non-Smad signals has been implicated in epithelial-mesenchymal transition (EMT) (Mihira et al 2012), this point should be checked more carefully. For instance, the authors analyzed cdc42 SmallGTPase. I expect that Rac1 could be involved as well (at least in vitro). In any case, I would like to know if your results are in line with the works mentioned above. Does small GTPase activation occur in the host (cannibal) cell (florentini) or in the "entotic cell" (Overholtzer)?

Last point: the biological relevance of the proposed mechanism of cell cannibalism. The authors say: "Nupr1-depleted cells accomplish more spontaneous cannibalism than control cells without treatment (7.1{plus minus}0.8 vs. 4.4{plus minus}0.3, respectively, as measured by FACS)". With these numbers more prudent statements should be provided.

Minor points

The authors included a Supplementary Figure (figure 6) to show the results obtained with other cell lines. These results are only mentioned in the discussion. I think that these data should merit a specific paragraph in the results section or they can be withdrawn. As it is, this supplementary figure is unconvincing.

The results obtained by FACS analysis are very interesting and the methodology seems appropriate. However, this represents a novel approach to the study of cell cannibalism and should be described in more detail (providing possible pitfalls as supplementary material). For example: does the dye diffuse after cell staining? In addition, the authors should show appropriate negative controls (for instance samples incubated at 4{degree sign}C).

Referee #2:

In the manuscript entitled "Cell cannibalism, a cell death process driven by the....." by Cano and colleagues, the authors explore an interesting cellular phenomenon that has been seen in pancreatic cancer and other tumors and explore the molecular mechanisms behind this entosis/cannibalism. Their data implicates a pathway involving TGFb and decreased Nupr1 in the regulation of this process. This paper is quite interesting and the topic of entosis is relatively new and would be of interest to the scientific and medical communities. Overall, the experiments are well done and the data justifies the conclusions. There are a few points that would improve the quality of the paper.
-The correlation of pathological/molecular findings to clinical outcome is always tricky, particularly in a relatively small cohort of heterogeneously treated patients. I would assume that since the correlation to survival was not reported, that this was not significant? I am also not clear on the

catergorization of metastasis. At some point, the overwhelming majority of pancreatic cancer patients will develop metastasis and the frequency of this will depend on interval and duration of followup, how they are followed (scans vs tumor markers), treatment, and many other confounding variables. Given this, it would seem that the only way to make a meaningful correlation would be the initial presentation of whether there are metastases or not (even this is problematic, but probably less so than looking at metastases at any point of treatment). I think this is a minor point of the paper and it should either be removed or significantly downplayed in the results/discussion.
-The description of pancreatic cancers acquiring a phagocyte-type phenotype characterized by weak CD68 staining is overstated. It would be helpful to use other Mac markers as well.
-to help rule out RNAi off-target effects, some of the Nupr1 siRNA experiments should be performed with a second siRNA
-If you performed an unbiased transcriptomic analysis (GSEA for example), are the phagocytosis gene sets significantly changed in response to Nupr1 KD.
-The cell-based studies are nicely done. The data would be strengthened if the authors looked at a collection of pancreatic cancer cell lines and characterized them for their basal levels of Nupr1. Presumably, the basal cannibalism levels would differ depending on Nupr1 expression - eg a low Nupr1 expressing line should have more basal cannibalism than Panc1. Likewise, elevated TGFb signaling in particular lines should show increases cannibalism. This would help substantiate the authors claims. Along these lines overexpression of Nupr1 should inhibit the TGFb induced cannibalism according to the model.
-Were there any phenotypic differences in the genetic model of pancreatic cancer when Nupr1 was knocked out? It seems strange to introduce these findings in this study, but state that they will be published elsewhere.

1st Revision - Authors' Response 11 May 2012

Point-by-point response to the reviewers

***** Reviewer's comments *****

Referee #1:

In this study Cano et al. performed an extensive series of cellular, biochemical, and molecular analyses on cell cannibalism in cancer. In particular, they carefully analyze cell cannibalism in pancreatic adenocarcinoma. Both histopathological analyses and in vitro studies have been carried out. The authors found pictures of cell cannibalism (also called "cell in cell") in samples from patients with pancreatic cancer. The authors also found that patients with pancreatic adenocarcinoma whose tumors display cell cannibalism develop less metastasis than those without. Several in vitro analyses have been performed in order to investigate the mechanisms underlying cell cannibalism. At least in vitro, cell cannibalism was found enhanced by TGFbeta and repressed by the Nuclear protein 1 (Nupr1) in pancreatic cancer cells. The authors conclude suggesting that inactivation of Nupr1 provokes a genetic reprogramming in PDAC cells that elicit cell cannibalism-associated cell-death in vitro and in vivo and that this behavior could exert a metastasis suppressor activity. Finally, the authors also suggest the potential implication of cell cannibalism (its induction) as a target in anticancer therapy.

I found this work very interesting and clearly written. It contains a number of very interesting findings, including some possible mechanism leading to cell cannibalism in pancreatic cancer cells. However, I have some points that must be considered and discussed in detail.

Major points

The major point is a general and conceptual issue. The idea that cell-in-cell (i.e. entosis) could be considered here is puzzling. The idea of these authors, if I have well understood, is that a small percentage of cancer cells are capable of engulfing and cannibalize their siblings. The authors seem to consider cell cannibalism as an "active" process of phagocytosis by non-professional phagocytes. This means that this is not entosis. Cell cannibalism was described several years ago by different authors (e.g. Steinhaus J. Virchows Arch 1891; Stroebe H. Beitrage Pathol 1892; and more recently: DeSimone et al 1980), but several further works have been carried out on this matter in different models (e.g. Mormone et al. 2006; Matarrese et al. 2011; Lai et al. 2010). A more careful analysis of literature must be included in this work). Authors please comment carefully. It is very important to discriminate between entosis or cannibalism.

Response 1/ We thank the reviewer for this insightful comment. Indeed, we have found that PDAC cells are able to cannibalize their siblings in a homotypic cell cannibalism (HoCC) process that is dependent on the inactivation of Nupr1, and the concomitant upregulation of phagocytosis-related genes in the cannibal cell. This means that what we see is driven by the cannibal cell and, thus, it is not entosis (i.e. an invasion of one cell into another one triggered by adhesion loss). We have more carefully introduced and discussed the diversity of cell-in-cell phenomena, including entosis, observed by us and others, as required by the reviewer. Moreover, additional experiments (included in Fig. 5) show that the homotypic cell cannibalism described here in PDAC cells does not share molecular requirements with entosis, strongly supporting the difference between these processes. Therefore, as suggested by the reviewer, we have added to the resubmitted manuscript a carefully crafted narrative on the differences between these phenomena.

Although clinical significance of cell cannibalism in cancer still remains unclear, there is a plethora of works that should be considered (cited) and discussed in this manuscript (among these Kojima et al. Acta Cytol 1998; Kumar et al. Acta Cytol 2001; Matyarrese et al 2008; Gupta et al 2003; Malorni et al. 2007; Abodief et al. 2006). These works suggest that, at variance with the hypothesis raised by these authors, cell cannibalism could represent: i) a cell survival mechanism for host cell and ii) a "negative" prognostic factor. This sounds very trustworthy. Authors please comment.

Response 2: We have addressed the reviewer concern by both providing new data as well as discussing and citing the articles pointed by the reviewer. To address the role of homotypic cell cannibalism upon starvation, we cultured PDAC in nutrient-free medium which function as an apoptotic stimulus for these cells (see Hamidi et al. JCI 2012 May 8) and assessed the percentage of cannibal cells. The new Figure 5e shows that homotypic cell cannibalism is not significantly induced in PDAC cells in these conditions, leading us to conclude that this process is not associated with survival upon starvation in pancreatic cancer cells. Consistently, we have recently shown that Nupr1 promotes PDAC survival under starvation (Hamidi et al.), while we show here that it represses homotypic cell cannibalism.

Regarding the role of cannibalism as a “negative prognostic factor” for this cancer, our results differ from those reported by Kojima et al. 1998; Kumar et al. 2001; Gupta et al 2003; and Abodief et al. 2006) performed in other cancers, leading us to conclude that: 1. Either homotypic cell cannibalism, like other “cell-eating-cell” processes (e.g: autophagy) can have different physiological and/or pathophysiological outcomes depending of the tumor type or that 2. the differences observed may reflect, the nature of the samples analyzed, which in our case (solid pancreatic tumors) precludes detachment-induced entosis, whereas in the studies cited (urine and other cancer effusions) entosis and other types of cell-in-cell phenomena cannot be ruled out. Thus, due to the originality and biomedical significance of our observations, we have carefully discussed these points in the revised version of the manuscript.

It could be hypothesized that pancreatic cancer could differ from other forms of cancer and/or that gemcitabine treatment could exert an inhibitory effect on cell cannibalism. Please check (in vitro).

Response 3: Following the reviewer's request, we tested the effect of gemcitabine on homotypic cell cannibalism in PDAC cells. The new supplementary figure 3, demonstrates that gemcitabine induces homotypic cell cannibalism in PDAC cells to levels comparable to Nupr1-knockdown. These results are totally congruent with our previous work showing that Nupr1 expression is indeed inhibited by gemcitabine (Giroux et al., Clin Cancer Res, 2006). Thus, the current data further support the existence of a negative correlation between the induction of cannibalism and Nurp1 expression, a process that is recapitulated by gemcitabine treatment.

The authors found "a strong relationship between cannibalism and metastasis suggesting an anti-metastasis role of cell-in-cell structures". I am not convinced by the power of this analysis based on very few patients. More prudent statements should be provided.

Response 4: It is a reasonable remark that this statement should be reformulated. It now stands "indicating an inverse relationship between cannibalism and metastasis and suggesting an anti-metastasis role of cell-in-cell structures".

In a previous work Cano and coauthors (2011) hypothesize that Nupr1 could modulate autophagy. The authors should evaluate some autophagic marker in their study. In fact, some papers in literature hypothesize a cross-talk between autophagy and cannibalism. I saw in literature that under starvation cell cannibalism might increase (more than inactivation of Nupr1 can do). Please check.

Response 5: As mentioned above, we did not observe a significant induction of homotypic cell cannibalism in PDAC cells upon starvation. In contrast, knockdown of ATG5 (which is necessary for both autophagy and entosis as the reviewer points out) did enhance homotypic cell cannibalism, indicating that autophagy and homotypic cell cannibalism do not overlap for PDAC survival under metabolic stress.

A further important point concerns the ability to engulf other cells. The authors say: "engulfed cells may be viable cells". Literature on the argument is very poor. However, as suggested by Lugini et al (2006) metastatic melanoma cells are capable of engulfing and digest live autologous CD8 lymphocytes. It could be of great interest to know if pancreatic cells do the same. On the basis of the results reported by the authors, I expect that these cells could not be able to cannibalize live lymphocytes that should kill them. This could at least partially explain the disparity with literature (in terms of prognosis).

R6/ This is a very interesting point. In order to shed light into this question, we reproduced the assay of Lugini et al. using PDAC (Panc-1) cells with or without Nupr1-knockdown and TGFβ treatment. We found that PDAC cells can engulf lymphocytes to levels comparable to melanoma cells (supplementary figure 6). We also found this ability to perform heterotypic-cannibalism to be independent of Nupr1 expression and insensitive to TGFβ. Thus, we conclude that the mechanism underlying homotypic cell engulfment by PDAC cells is different to the previously described in melanoma. Hence, for our histological examinations, we only focused on cell-in-cells involving tumors cells exclusively, and excluded those containing lymphocytes or polynuclear cells. Therefore, our results are not contradictory to those presented by Lugini et al.

A further important point concerns cytoskeleton involvement in the cell cannibalism. The proposed model (Nupr1-inactivation, TGFbeta effects) seems to represent a useful model to investigate host cell cytoskeleton. In particular, it could be very interesting to evaluate Rho family small GTPase in the system. In fact, at least two papers (Fiorentini et al 2001; Overholtzer et al. 2007) hypothesize a role for these proteins in cytoskeleton dynamics and cell engulfment. Since activation of Rho signals via TGF-beta-induced non-Smad signals has been implicated in epithelial-mesenchymal transition (EMT) (Mihira et al 2012), this point should be checked more carefully. For instance, the authors

analyzed cdc42 Small GTPase. I expect that Rac1 could be involved as well (at least in vitro). In any case, I would like to know if your results are in line with the works mentioned above. Does small GTPase activation occur in the host (cannibal) cell (florentini) or in the "entotic cell" (Overholtzer)?

Response 7: This point was a common question for reviewers 1 and 3. Entosis was indeed found to be dependent on cytosqueletton rearrangement driven by a ROCK1-dependent Rho-GTPase activity (Overholtzer et al. 2007). Therefore, we addressed the question of whether Rock1 was also a key effector of homotypic cell cannibalism. On the contrary to entosis, we found that Rock1 inactivation rather enhanced HoCC. Hence, homotypic cell cannibalism and entosis do not share the mechanistic of cytoskeleton rearrangement. Note that this data further address other important questions posed by the reviewer (see response to concern 1)

Last point: the biological relevance of the proposed mechanism of cell cannibalism. The authors say: "Nupr1-depleted cells accomplish more spontaneous cannibalism than control cells without treatment (7.1{plus minus}0.8 vs. 4.4{plus minus}0.3, respectively, as measured by FACS)". With these numbers more prudent statements should be provided.

Response 8: Basal level of cell cannibalism is rather low in Panc-1 cells, which makes difficult to conclude on the effect of Nupr1 inhibition without any treatment. More convincing evidence that high Nupr1 expression is inhibitory for homotypic cell cannibalism in PDAC cells is now provided in figure 5d. After the recommendations of reviewer 2, we performed a wider screening of cell cannibalism in different human PDAC cell lines without TGFβ treatment and matched the rate of homotypic cell cannibalism to the level of Nupr1 transcript (measured by qRT-PCR). In congruency with other dataset from this study, these experiments reveal an inverse correlation between the level of Nupr1 transcript and induction of homotypic cell cannibalism (see response to concern 3).

Minor points

The authors included a Supplementary Figure (figure 6) to show the results obtained with other cell lines. These results are only mentioned in the discussion. I think that these data should merit a specific paragraph in the results section or they can be withdrawn. As it is, this supplementary figure is unconvincing.

R9/ Following the reviewer's remark, we decided to focus on the data on PDAC in this manuscript. This figure was removed from the revised version.

The results obtained by FACS analysis are very interesting and the methodology seems appropriate. However, this represents a novel approach to the study of cell cannibalism and should be described in more detail (providing possible pitfalls as supplementary material). For example: does the dye diffuse after cell staining? In addition, the authors should show appropriate negative controls (for instance samples incubated at 4{degree sign}C).

Response 10: We thank the reviewer for the comments on our innovative FACS-based quantification method. As requested, we have provided a detailed description of the methods using both fluorescent constructs and dyes. Briefly, for these experiments, fluorescent cells were obtained by stable transduction with lentiviral EGFP- or DsRed-expressing vectors. For experiments using dyes, the CFSE and CMPTX dyes used are not fluorescent at first, and are metabolized into fluorescent products that cannot exit the cell. For these experiments, cells were stained, washed and cultured overnight, then washed again in order to discard any residual dye before mixing green- and

red-labeled cells. Regarding incubation of cells at 4°C, assessment of homotypic cell cannibalism requires an incubation of 48 h, which is far too long for cells to survive at 4°C.

Referee #2:

In the manuscript entitled "Cell cannibalism, a cell death process driven by the....." by Cano and colleagues, the authors explore an interesting cellular phenomenon that has been seen in pancreatic cancer and other tumors and explore the molecular mechanisms behind this entosis/cannibalism. Their data implicates a pathway involving TGFb and decreased Nupr1 in the regulation of this process. This paper is quite interesting and the topic of entosis is relatively new and would be of interest to the scientific and medical communities. Overall, the experiments are well done and the data justifies the conclusions. There are a few points that would improve the quality of the paper.

-The correlation of pathological/molecular findings to clinical outcome is always tricky, particularly in a relatively small cohort of heterogeneously treated patients. I would assume that since the correlation to survival was not reported, that this was not significant? I am also not clear on the categorization of metastasis. At some point, the overwhelming majority of pancreatic cancer patients will develop metastasis and the frequency of this will depend on interval and duration of follow-up, how they are followed (scans vs tumor markers), treatment, and many other confounding variables. Given this, it would seem that the only way to make a meaningful correlation would be the initial presentation of whether there are metastases or not (even this is problematic, but probably less so than looking at metastases at any point of treatment). I think this is a minor point of the paper and it should either be removed or significantly downplayed in the results/discussion.

Response 1: We agree with the reviewer that it is difficult to satisfyingly comment and document on the outcome of PDAC patients, in particular when one considers the fact that samples available for this type of analysis originate from a small fraction of PDAC patients who can receive surgery. As the reviewer correctly pointed out, at some point most PDAC patients will develop metastasis, and this stresses even more the failure of available treatments to fight the disease. For these reasons, we applied strict standardized criteria to our cohort of PDAC patients regarding eligibility for surgery, which include absence of metastasis at the time of resection, thus providing us with a comparable start point. We do find a statistically significant reduction of metastasis formation among patients with homotypic cell cannibalism. More than revealing a prognosis factor for PDAC, what we believe that our study reveals a phenomenon (homotypic cell cannibalism) that, because it leads to cancer cell-death, could be worth targeting for therapeutical purpose. Thus, we remain optimistic that the Nupr1-regulated pathway described here will fuel research in this biomedical relevant direction.

-The description of pancreatic cancers acquiring a phagocyte-type phenotype characterized by weak CD68 staining is overstated. It would be helpful to use other Mac markers as well.

Response 2: We agree with the reviewer that the sole CD68 staining is insufficient to conclude on a phagocytoid phenotype, and we did not mean this. For this reason, in page 6 of the current manuscript, we carefully described our data as follow: "our results suggested that PDAC epithelial cells may acquire a phagocyte-like phenotype characterized by the ectopic expression of CD68 and the ability to cannibalize". This hypothesis is supported by our experiments, confirming that the ability of PDAC cells to cannibalize their siblings is accompanied by an up-regulation of a number of phagocytosis-related genes upon Nupr1-depletion, including CD68. Our results are congruent with other recent studies which correlate CD68 expression with cell-in-cells figures (Fernandez-Flores A. Rom J Morphol Embryol. 2012;53(1):15-22; McKenna M. J Clin Pathol. 2008 May;61(5):648-51). We have followed the reviewer advice as it relates to measuring other markers. However, we did not detect modifications of Mac-1 expression after Nupr1-deletion in Panc-1 cells, and this transcript was barely detected in Panc-1 cells. Consistently, we did not detect Mac-1

staining in cancer cells of cannibal cell-containing human PDAC samples (our unpublished data). Thus, we are confident that the description of the data faithfully represents our observations of the phenotype observed in Nupr1-depleted cannibal cells.

-to help rule out RNAi off-target effects, some of the Nupr1 siRNA experiments should be performed with a second siRNA

Response 3: This is a pertinent remark. We have now used two additional Nupr1-specific siRNAs (siNupr1-1 and siNupr1-2) which yield identical results (Supplementary figure 5).

-If you performed an unbiased transcriptomic analysis (GSEA for example), are the phagocytosis gene sets significantly changed in response to Nupr1 KD.

Response 4: The reviewer's demand for an unbiased transcriptomic analysis is a sensible question. In this regard, our approach presented in the manuscript consisted on a naïve interrogation of the microarray data based on the cited Gene Ontology lists. This approach was fruitful, since it revealed a set of up-regulated genes after Nupr1-depletion in PDAC cells from which, CDC42, CXCL1 and CXCL6 were found to be required for TGFβ-induced homotypic cell cannibalism (Fig. 6). On the reviewer's demand, we performed GSEA and GO-ANOVA analysis on our set of myeloid-related genes. While GSEA analysis did not yield a very nice enrichment ($p=0.1$), GO-ANOVA revealed a supplementary set of myeloid-related genes to those we first detected. Supplementary Table 4 recapitulates the GO-ANOVA data. Thus, while Nupr1-depleted cannibal cells up regulates phagocytosis genes, which may elicit to homotypic cell cannibalism, the gene expression profile does not support a full transdifferentiation of PDAC cells into professional phagocytic (myeloid) cells. This data has been appropriately discussed in our manuscript (see response to concern 2)

-The cell-based studies are nicely done. The data would be strengthened if the authors looked at a collection of pancreatic cancer cell lines and characterized them for their basal levels of Nupr1. Presumably, the basal cannibalism levels would differ depending on Nupr1 expression - eg a low Nupr1 expressing line should have more basal cannibalism than Panc1. Likewise, elevated TGFb signaling in particular lines should show increases cannibalism. This would help substantiate the authors claims. Along these lines overexpression of Nupr1 should inhibit the TGFb induced cannibalism according to the model.

Response 5: Following this helpful remark of the reviewer, we added to the revised manuscript the assessment of homotypic cell cannibalism and Nupr1-transcript expression in human pancreatic cancer cell lines (Capan-1, Capan-2, PATU8988T, PATU8902, MiaPaCa2 and Panc-1) that is depicted in Figure 5d. The results of these experiments reinforced our previous conclusion that high levels of Nupr1 expression are inhibitory for homotypic cell cannibalism and vice versa. Consistently, we found that overexpression of Nupr1 in Panc-1 cells using a lentiviral vector (pCCL-Nupr1) inhibited homotypic cell cannibalism compared to cells transduced with an empty vector (Figure 5d, right).

-Were there any phenotypic differences in the genetic model of pancreatic cancer when Nupr1 was knocked out? It seems strange to introduce these findings in this study, but state that they will be published elsewhere.

Response 6: We thank the reviewer for his/her consideration since, as suggested, this type of data will indeed soon be published elsewhere, and we would like to keep it confidential by now. However, we can comment that the Nupr1-ko affects several hallmarks of tumor progression

including cancer cell proliferation, apoptosis, EMT and the homotypic cell cannibalism reported here. In addition, concomitant deletion of Nupr1 and Ink4a genes in the pancreas also affect the mice viability. This careful, laborious, and resource consuming studies are by their nature beyond the scope of the current manuscript.

Referee #3:

Cannibalism is a cell-in-cell formation by entosis. Several recent studies have suggested the role of cannibalism in an array of cellular activities ranging from aneuploidy to nonapoptotic cell death. Cano et al., aim to address the role of cannibalism in pancreatic cancer metastasis. Although this study is of great interest to better understanding cannibalism process and exhibits potential in intervention of pancreatic cancer progression, the current study is rather preliminary and additional works should be carried out to validate their claims. In short, I am supportive for its publication, pending the following issues being addressed satisfactorily:

1. The regulatory mechanisms underlying of TGF-beta and nuclear protein 1 remain elusive after this study. It is unclear how the opposing effect of TGF-beta and Nup1 is orchestrated in pancreatic cells. Mechanistic connection of Nup1 to entosis in pancreatic cancer should be elucidated;

Response 1/ This is a very interesting remark. We have shown previously that TGFβ stimulates Nupr1 expression and that, in turn, Nupr1 enhances Smad-dependent transcription (Malicet et al, 2006). We show here evidence that this activation participates to proper EMT of PDAC cells (Fig.3), which is expected to enhance tumor progression. Consistently, Nupr1 is overexpressed in PDAC and was found to be inversely correlated to apoptosis (Su, S. B., et al. 2001. Clin Cancer Res 7(5): 1320-1324). The results shown in this manuscript demonstrate that: 1) PDAC cells are able to perform homotypic cell cannibalism, 2) this phenomenon occurs at low frequency in human PDAC (3.5±0.8%, page 5), 3) in vivo, it only occurs in cells that do not express Nupr1 and, 4) low levels of Nupr1 expression result in enhanced homotypic cell cannibalism (Fig. 5). Therefore, we have come to a hypothetical model that is exposed in the discussion section. Briefly, in most PDAC cells that strongly express Nupr1, TGFβ will induce EMT and will detach and migrate to form metastasis. In the case where a genetic or an epigenetic event inactivates Nupr1 expression, a PDAC cell stimulated by TGFβ would be freed to express phagocyte-related genes (e.g. CD68, CDC42 and CXCL1) and become inclined to HoCC-associated cell death. Regarding entosis, the new data presented in figure 5e allowed us to distinguish from homotypic cell cannibalism in terms of molecular requirements, as exposed previously in the responses to reviewers 1 and 2.

2. The real-time cannibalism should be imaged so the effects of TGF-beta and Nupr1 can be distinguished;

Response 2/ This point was very difficult to address with the tools available in our lab. In fact, entosis takes 2-3 hours as reported by Overholtzer et al. (easy to be imaged), whereas TGFβ-induced HoCC in Nupr1-depleted PDAC cells cannot be detected before 48 h, likely due to the fact that changes in the gene expression profile must be established. Unfortunately, our time lapse equipment does not support the optimal conditions (5% CO_2) allowing siNupr1-transfected cells to survive for the entire time required for homotypic cell cannibalism. A motion of this phenomenon would definitely be a beautiful educational asset, although it may not be helpful discriminating between the effects of TGFβ and Nupr1 in this process. Indeed, as exposed above, Nupr1 is likely to act as a self-regulatory brake to TGFβ-induced homotypic cell cannibalism. In this case, Nupr1-depletion would be a release from the brake and imaging would not add information to the FACS-data.

3. It has been established the ROCk1 participates in entosis in several tumor models. The authors should address the role of ROCK1 in pancreatic tumor cannibalism;

Response 3/ We have performed experiments to test the involvement of ROCK1 in homotypic cell cannibalism, by the use of specific siRNA-mediated knockdown. Our results demonstrate that, contrary to entosis, ROCK-1 depletion enhanced homotypic cell cannibalism in PDAC cells (Figure 5e). Hence, it appears that cytosqueleton remodeling in HoCC is not driven by a ROCK1-mediated pathway but by a cdc42-dependent one.

4. Is the entosis in pancreatic cancer dependent on E-cadherin?

Response 4/ We tested the impact of E-cadherin (CDH1) inactivation on homotypic cell cannibalism in PDAC cells. As shown in figure 5e (right), homotypic cell cannibalism was not affected by E-cadherin-depletion. Thus, in contrast to entosis, it does not appear to be involved in homotypic cell cannibalism. This is congruent with the rest of our cell biological datasets showing that TGFβ promotes homotypic cell cannibalism, while it represses E-cadherin expression.

Together, the results obtained by addressing the concerns 4 and 5 from the reviewer demonstrating that ROCK1 and E-cadherin are not pivotal for homotypic cell cannibalism, along with the observation that this process is dependent on Nupr1-depletion and upregulation of phagocytosis-related genes *in the cannibal cell*, indicate that the homotypic cell cannibalism described here is not entosis. Moreover, entosis was shown to be a rapid process that occurs within a couple of hours after adhesion loss, whereas homotypic cell cannibalism required 48 h of incubation of PDAC cells with TGFβ, suggesting that these cells needed to undergo physiological transformations to perform cannibalism. In addition, the only factor shown to induce the cell-in-cell invasion process of entosis so far is adhesion loss, which is more likely to operate in effusions (e.g. ascites). Our finding of TGFβ as a promoting factor for HoCC is in the line of previous work showing that a serum-derived factor enhanced cell cannibalism and death of SCCL (small cell carcinoma of the lung) cells (Brouwer M, et al. Cancer Res 1984;44:2947-51), which again supports a tumor suppressive role for homotypic cell cannibalism.

2nd Editorial Decision 13 June 2012

Thank you for the submission of your revised manuscript to EMBO Molecular Medicine. We have now received the enclosed reports from the referees that were asked to re-assess it. As you will see the reviewers are now supportive and I am pleased to inform you that we will be able to accept your manuscript pending the following final editorial amendments:

1/ Data of gene expression experiments described in submitted manuscripts should be deposited in a MIAME-compliant format with one of the public databases. We would therefore ask you to submit your microarray data to the ArrayExpress database maintained by the European Bioinformatics Institute for example. ArrayExpress allows authors to submit their data to a confidential section of the database, where they can be put on hold until the time of publication of the corresponding manuscript. Please see http:www.ebi.ac.uk/arrayexpress/Submissions/ or contact the support team at arrayexpress@ebi.ac.uk for further information.

2/ Please only provide 5 keywords

3/ Please on the 1st page of your pdf file combining all the Supplementary Information, provide a Table of Content.

4/ Within the figure legend, we noticed that Figure 5 does not have a title. Please provide one.

5/ Ethical statements:

-For Research Articles and Reports submitted to EMBO Molecular Medicine reporting experiments on live vertebrates and/or higher invertebrates, the corresponding author must confirm that all experiments were performed in accordance with relevant guidelines and regulations. The manuscript must include a statement in the Materials and Methods identifying the institutional and/or licensing committee approving the experiments, including any relevant details.

-For experiments involving human subjects or human samples, the submission must include a statement that informed consent was obtained from all subjects and that the experiments conformed to the principles set out in the WMA Declaration of Helsinki [http://www.wma.net/en/30publications/10policies/b3/] and the NIH Belmont Report [http://ohsr.od.nih.gov/guidelines/belmont.html]. Additionally, authors must identify the institutional committee that approved the experiments. Any restrictions on the availability or on the use of human data or samples should be clearly specified in the manuscript.

Please submit your revised manuscript within two weeks. I look forward to seeing a revised form of your manuscript as soon as possible.

I look forward to reading a new revised version of your manuscript as soon as possible.

Yours sincerely,

Editor
EMBO Molecular Medicine

***** Reviewer's comments *****

Referee #2 (Comments on Novelty/Model System):

The authors used a nice combination of human specimens, cells lines and mouse models in this study

Referee #2 (Other Remarks):

The authors have addressed my concerns satisfatorily through text chnges as well as new data included in the manuscript.

Referee #3:

The authors have carried out a careful revision and addressed most of my concerns. It is now acceptable for this journal.
