# Supplementary material for: Homotypic cell cannibalism, a cell-death process regulated by the nuclear protein 1, opposes to metastasis in pancreatic cancer
Source: EMBO Mol Med. 2012 Jul 23;4(9):964–79. doi: 10.1002/emmm.201201255 (PMC3491828; doi:10.1002/emmm.201201255)
Supplement: Supplementary file 2 [file emmm0004-0964-SD2.pdf]

Supporting information

Homotypic cell cannibalism, a cell-death process regulated by the Nuclear Protein 1, opposes to metastasis in pancreatic cancer

Carla E Cano[1-4], María José Sandí[1-4], Tewfik Hamidi[1-4], Ezequiel L Calvo[5], Olivier Turrini[2], Laurent Bartholin[6-9], Céline Loncle[1-4], Véronique Secq[1-4], Stéphane Garcia[1-4], Gwen Lomberk[10], Guido Kroemer[11-15], Raul Urrutia[10], Juan L Iovanna[1-4$].

[1]Inserm U1068, CRCM, Cell Stress, Marseille, F-13009, France; [2]Institut Paoli-Calmettes, Marseille, F-13009, France; [3]Aix-Marseille University, F-13284, Marseille, France; [4]CNRS, UMR7258, CRCM, Marseille, F-13009, France; [5]Molecular Endocrinology and Oncology Research Center, CHUL Research Center, Quebec, Canada; [6]INSERM U1052, Lyon, F-69008, France; [7]CNRS UMR 5286, Lyon, F-69008, France; [8]Centre de Recherche en Cancérologie de Lyon, Centre Léon Bérard, Lyon, F-69008, France; [9]University of Lyon, Lyon, F-69003, France; [10]Laboratory of Epigenetics and Chromatin Dynamics, Gastroenterology Research Unit, Departments of Biochemistry and Molecular Biology, Biophysics, and Medicine, Mayo Clinic, Rochester, USA; [11]INSERM, U848, Villejuif, France; [12]Metabolomics Platform, Institut Gustave Roussy, Villejuif, France; [13]Centre de Recherche des Cordeliers, Paris, France; [14]Pôle de Biologie, Hôpital Européen Georges Pompidou, AP-HP, Paris, France; [15]Université Paris Descartes, Paris 5, Paris, France.

Table of content

Supplementary Figure legends

Supplementary Figure 1.

Supplementary Figure 2.

Supplementary Figure 3.

Supplementary Figure 4.

Supplementary Figure 5.

Supplementary Figure 6.

Supplementary Figure 7.

Supplementary Figure 8.

Supplementary Figure 9.

Supplementary Figure 10.

Supplementary Table 1.

Supplementary Table 2.

Supplementary Table 3.

Supplementary Table 3.

Supplementary Table 4.

Supplementary Figure legends:

<u>Supplementary Figure 1:</u> Cannibal cells in PDAC lack expression of Nupr1. Immunohistological staining of Nupr1 in human PDAC samples. a. Whole picture of the crop shown in Figure 1f. b, c and d. Several examples of cannibal cells observed in PDAC patients. e. Low magnification picture of a human PDAC sample displaying strong Nupr1 staining in epithelial cancer cells.

<u>Supplementary Figure 2:</u> HoCC in human PDAC cell lines. Dot plots illustrate a representative experiment that yielded the results depicted in figure 5d, showing FACS measurements HoCC in PDAC cells lines that were stained with CFSE or CMPTX dyes (Capan-1, Capan-2, PATU8988T, PATU8902, Panc-1, Panc-1-pCCL and Panc-1-pCCLNupr1), or stably transduced with fluorescent pCCL-GFP or pLVX-DsRed vectors, prior to mixing and incubation for 48 h at 37°C. For cells transiently stained with dyes, cannibal gates were designed based in dot blots from cells mixed just before FACS acquisition (0 h).

<u>Supplementary Figure 3:</u> Gemcitabine increases HoCC and inhibits Nupr1 expression in Panc-1 cells. a. EGFP- and DsRed-Panc-1 cells were mixed at equal numbers and cultured in the presence or absence of gemcitabine (GEM, 50 µM) for 48 h, then, % of cannibal cells was assessed by FACS. b. qRT-PCR assessment of Nupr1 expression in Panc-1 cells with or without GEM treatment for 24 h. Cyclophilin A mRNA expression was assessed as housekeeping control. Data are means of triplicates ± SEM.

<u>Supplementary Figure 4:</u> Effect of nutrient deprivation and inhibition of molecular actors of autophagy and entosis on HoCC. EGFP- and DsRed-Panc-1 cells were mixed at equal numbers, transfected with the indicated siRNAs, and cultured in nutrient-free EBSS medium or conventional medium without (Mock) or with TGFβ (10 ng/ml). Dot plots illustrate a representative experiment of the results depicted in Figure 5e.

<u>Supplementary Figure 5:</u> Nupr1-knockdown enhances HoCC. Nupr1-expression was knocked-down using two additional siRNAs that were reported previously (Giroux et al. 2006) (a). In b and c, histogram and dot plots of a representative experiment showing that additional Nupr1-specific siRNAs led to reproduction of results obtained using siNupr1. Data are means of triplicates ± SEM. * $p \leq 0.05$ compared to siCtrl.

<u>Supplementary Figure 6:</u> Nupr1-depletion does not affect xeno-cannibalism in PDAC cells. Panc-1 cells were transfected with siCtrl or siNupr1 48 h prior to mixing with CFSE-stained PBMC, and cultured with or without TGFβ for 6 h at the temperatures indicated. (left)

histograms show overlap of CFSE+ cell counts in the conditions indicated. (right) Bars show mean percentage of CFSE+ cells in each condition. Data are means of triplicates ± SEM.

<u>Supplementary Figure 7:</u> **Nupr1-knockdown allows upregulation of phagocytosis-related genes in Panc-1 cells.** a. qRT-PCR confirming enhanced or ectopic expression of phagocytosis-related genes in Nupr1-depleted cells compared to control cells, after 24 h of TGFβ1 treatment. Cyclophilin A mRNA expression was used as housekeeping control. Data are means of triplicates ± SEM. * $p \leq 0.05$, $ $p \leq 0.01$, £ $p \leq 0.005$, & $p \leq 0.001$. b. Heatmap showing altered expression of TGFβ-induced EMT-related genes in Panc-1 cells after Nupr1-knockdown (siNupr1) compared to controls (siCtrl).

<u>Supplementary Figure 8:</u> **FACS-based quantification of the impact of inactivation of phagocytosis related genes on HoCC.** Figure shows representative dot plots of experiments that yielded the results exposed in Figure 6b, demonstrating the impact of inactivation of: a) CCR1 expression, CXCL1 and CXCL6 activity (by the use of neutralizing antibodies); b) CCR1-mediated signal using the repertaxin (RTX) antagonist; c) CSF1 and IL-11 expressions; d) cdc42 expression.

<u>Supplementary Figure 9:</u> **p38MAPK activity, but not Smad4, is necessary for HoCC in Nupr1-deficient PDAC cells.** Dot plots of a representative experiment that yielded the results depicted in Figure 6e and f. a. EGFP- and DsRed-BxPC3 (Smad-4-mutated) cells were transfected with siCtrl ot siNupr1 and treated with or without TGFβ for 48 h; b. EGFP- and DsRed-Panc-1 cells were transfected with siCtrl ot siNupr1 and treated with or without TGFβ, and with or without the p38MAPK-inhibitor SB203580 for 48 h.

<u>Supplementary Figure 10:</u> **Nupr1-deficiency enables cell cannibalism and death in murine pancreatic tumors.** Low magnification confocal microscopy pictures of cleaved caspase-3 (red), cytokeratin-19 (KRT19, green) and the phagocyte marker F4/80(blue) immunofluorescent staining on histological sections of *Nupr1WT* and *Nupr1KO Pdx1-creER; LSL-Kras*^{G12D}*;Ink4A*$^{fl/fl}$ pancreatic tumors. The white box delimitates the region amplified in Figure 7b.

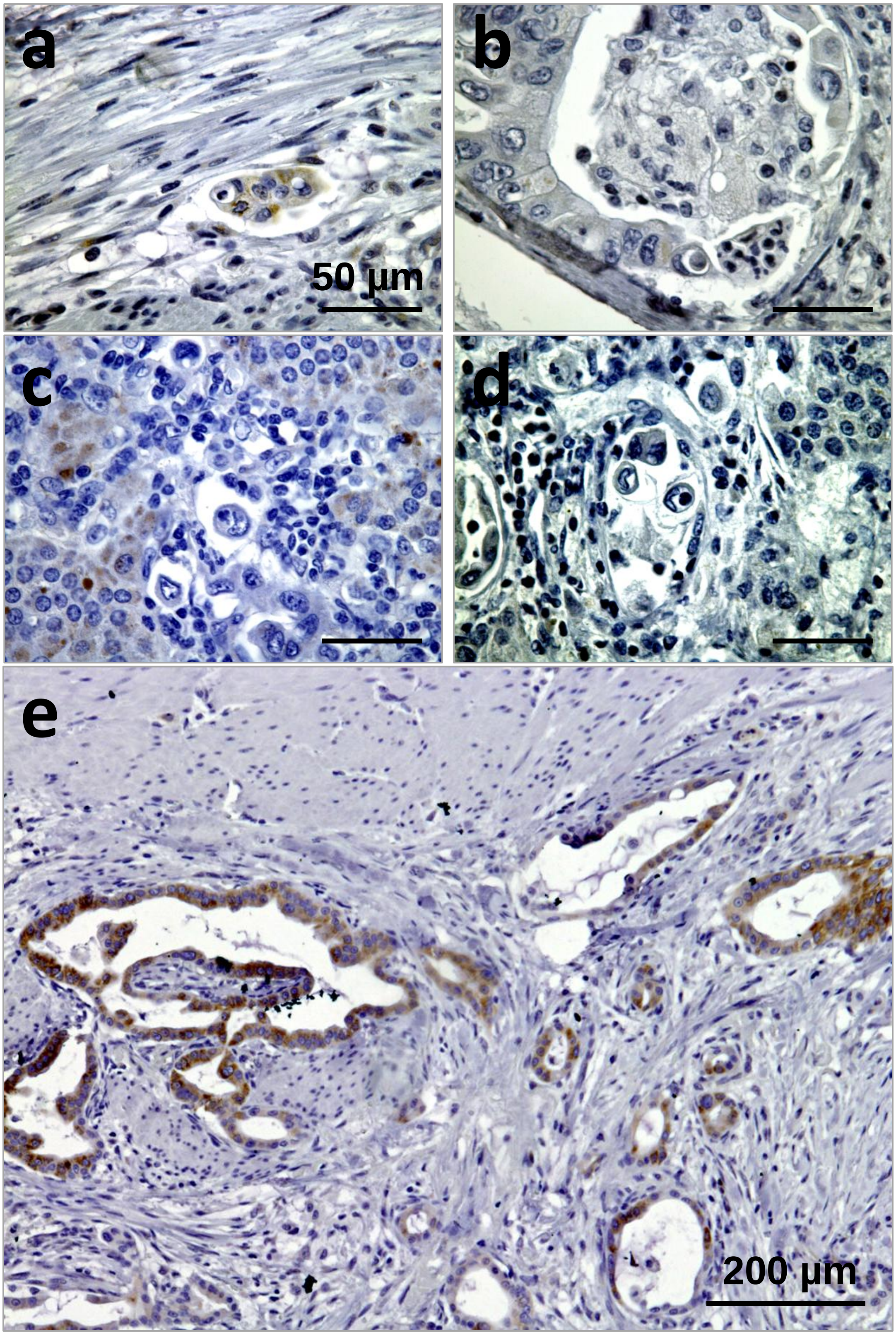

Supplementary Figure 1

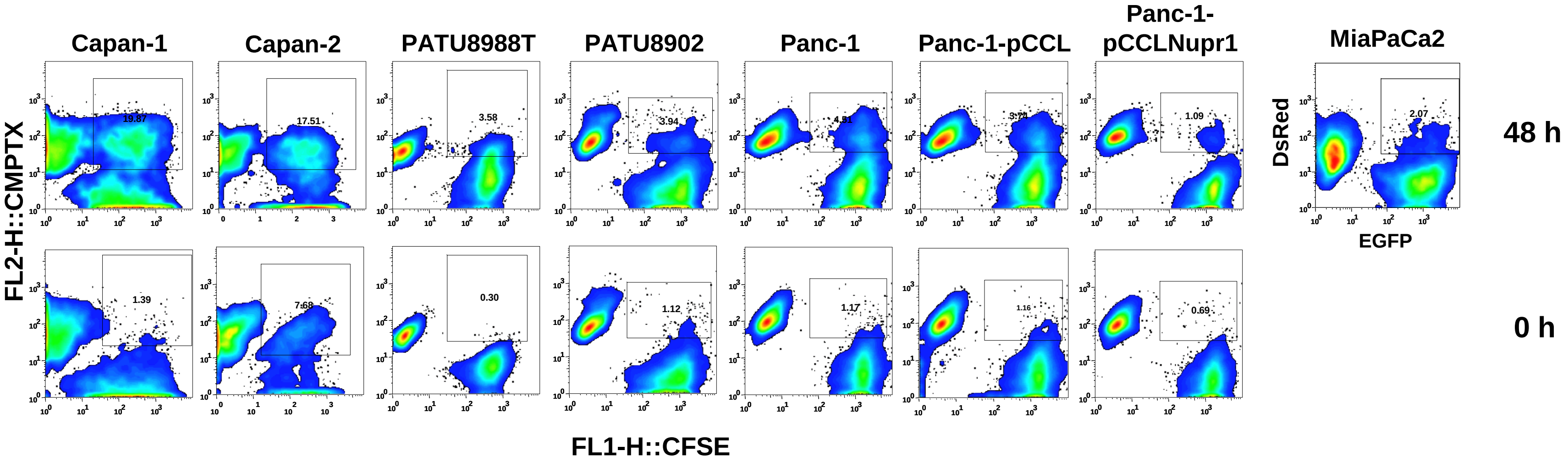

Supplementary Figure 2

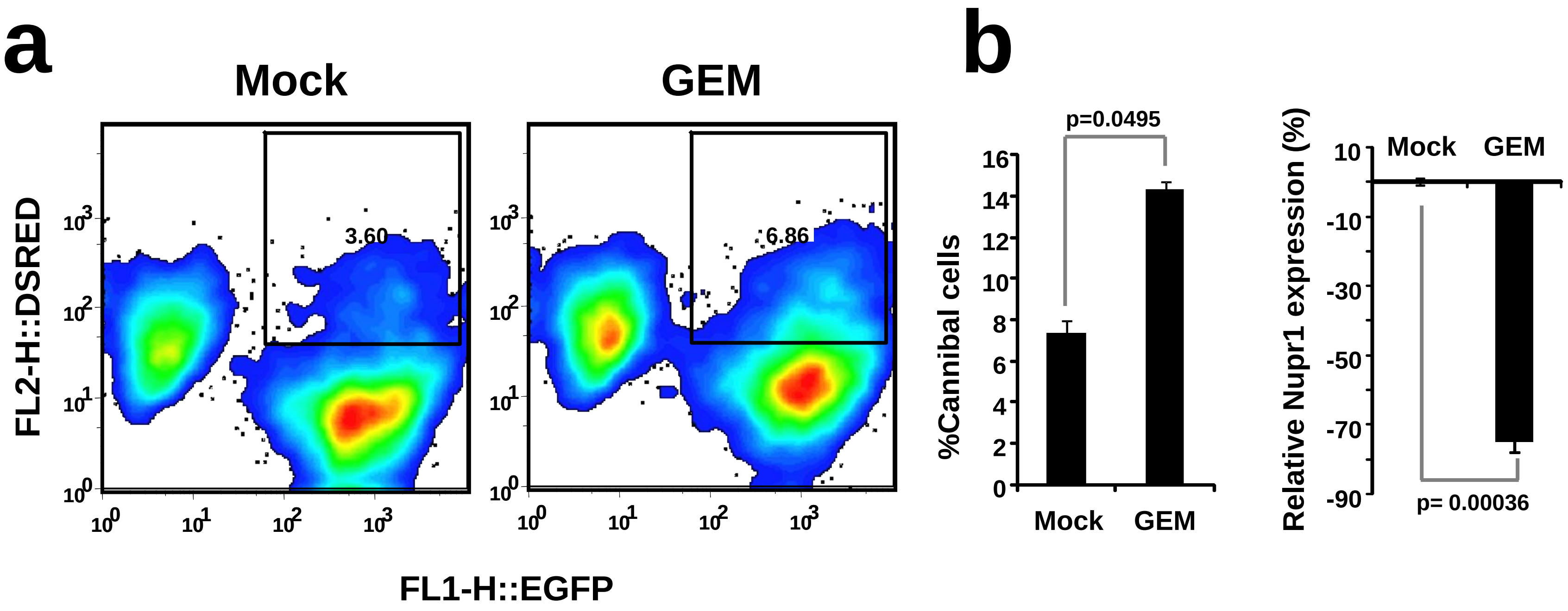

Supplementary Figure 3

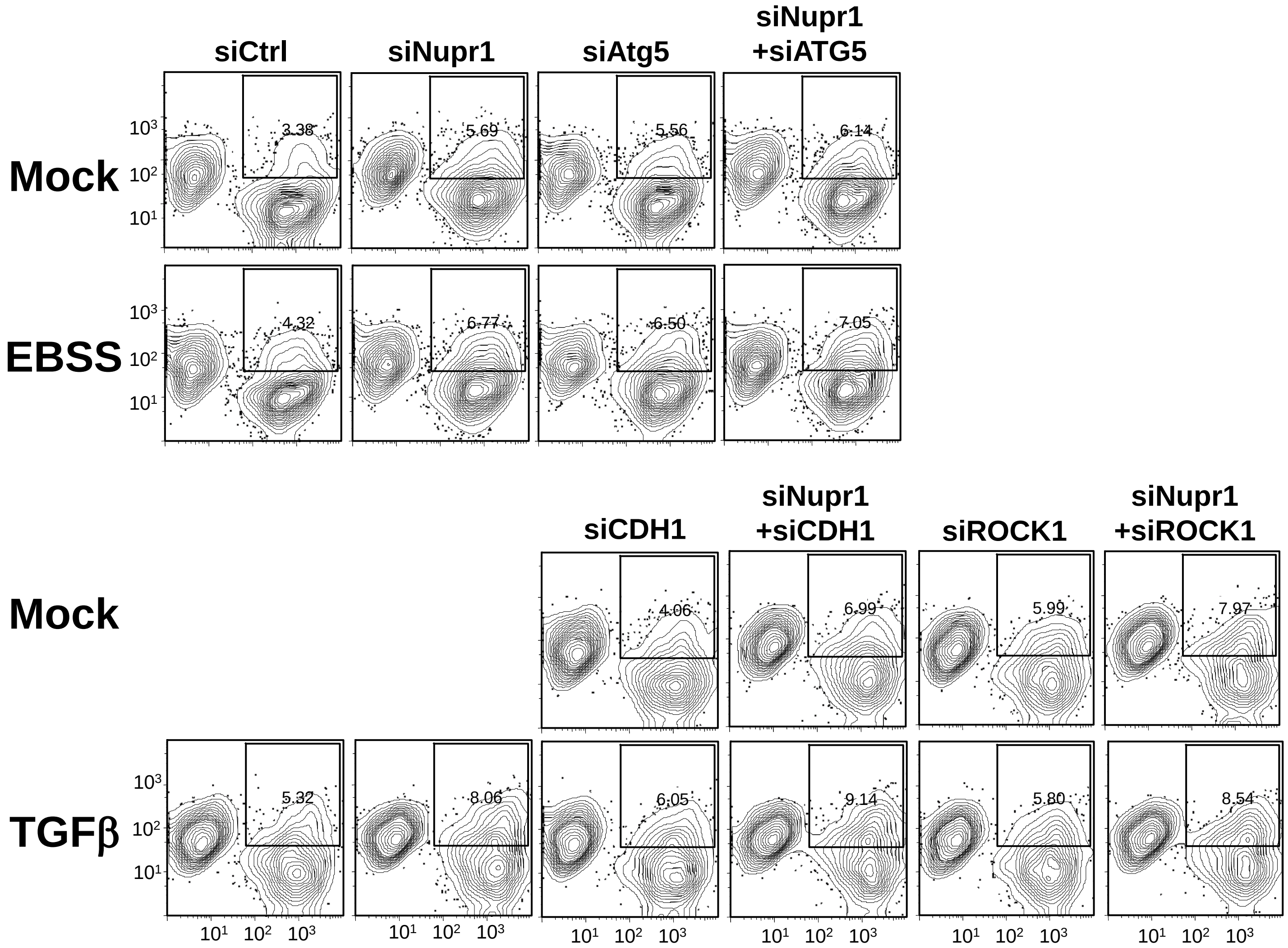

Supplementary Figure 4

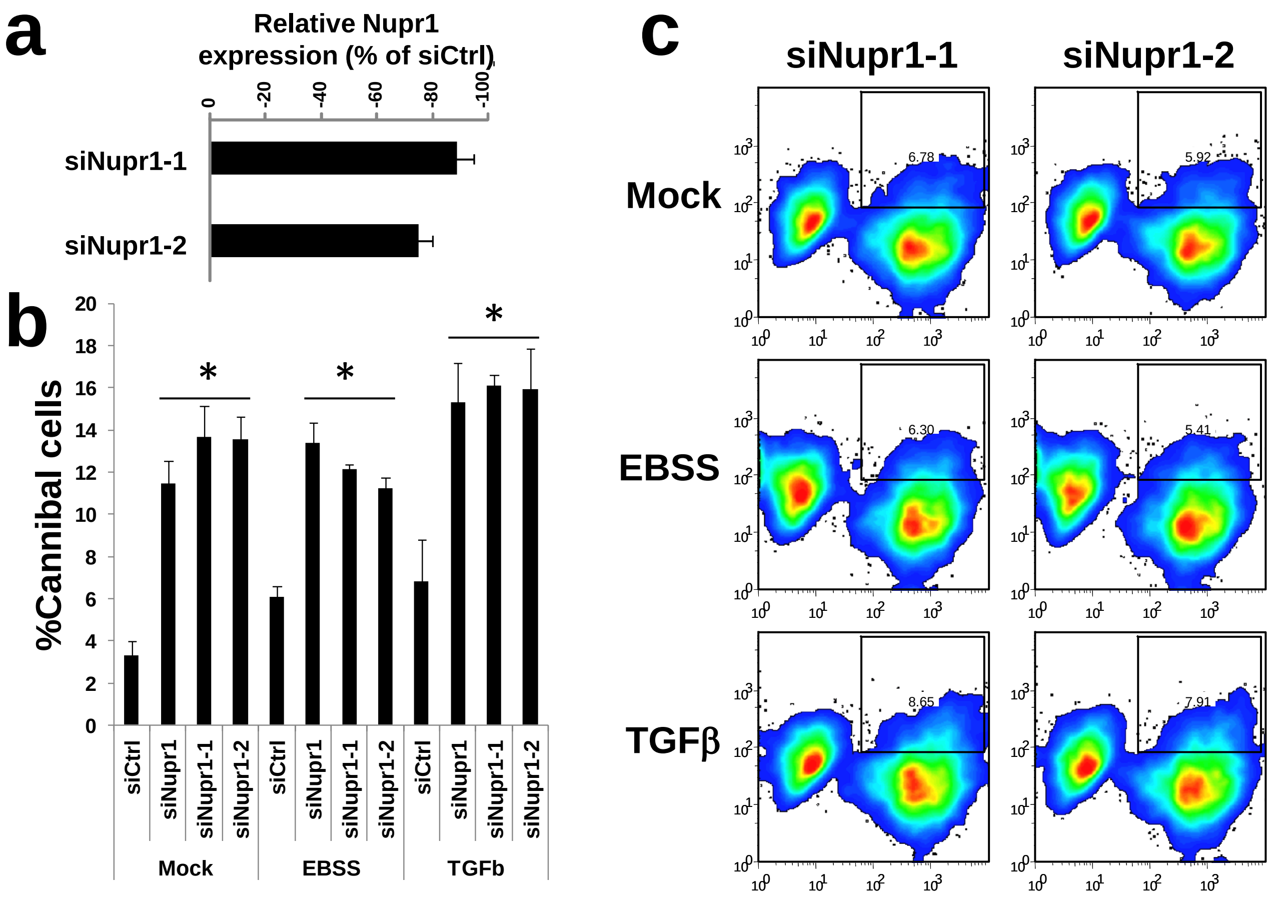

Supplementary Figure 5

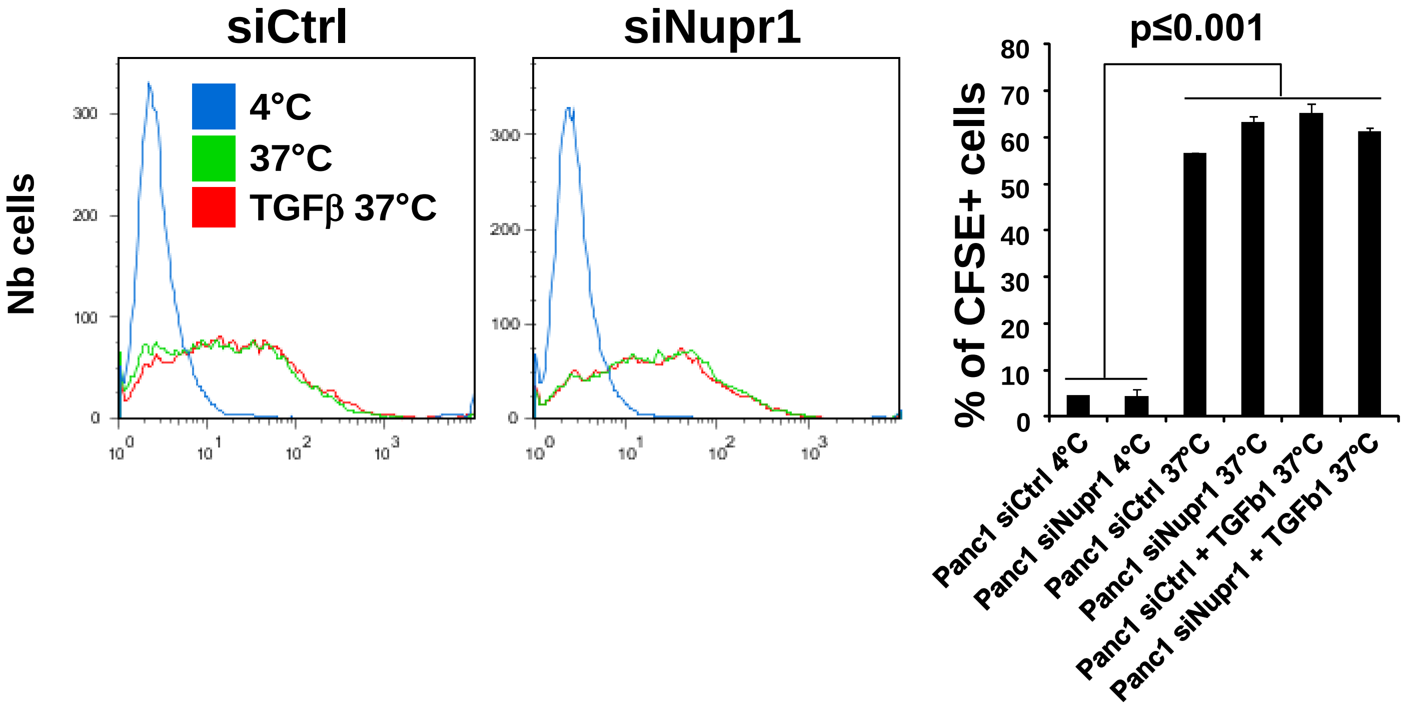

Supplementary Figure 6

a

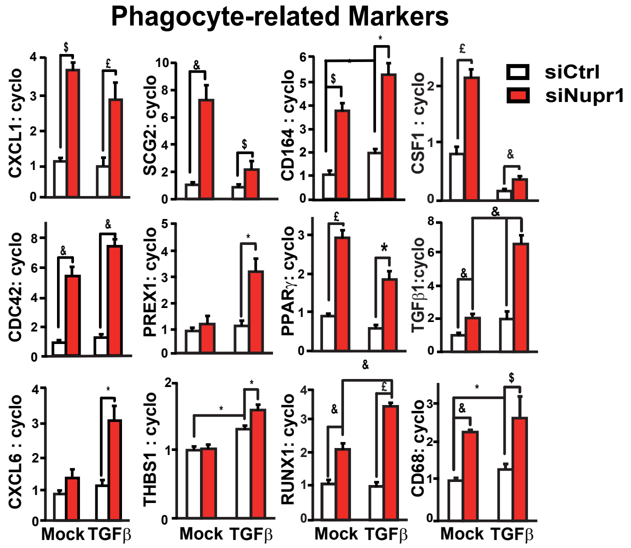

b

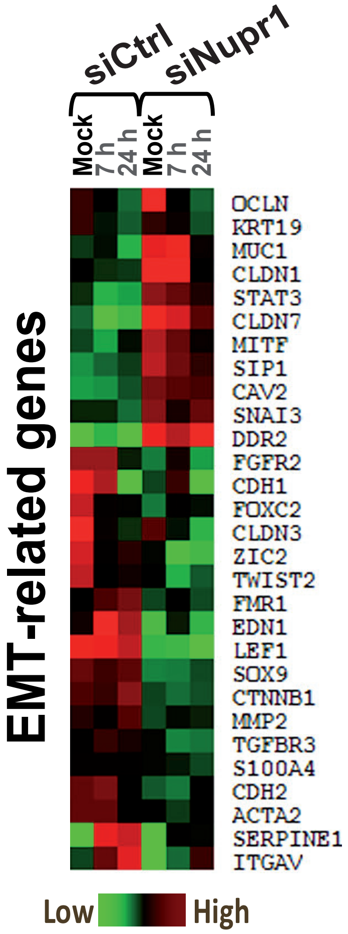

Supplementary Figure 7

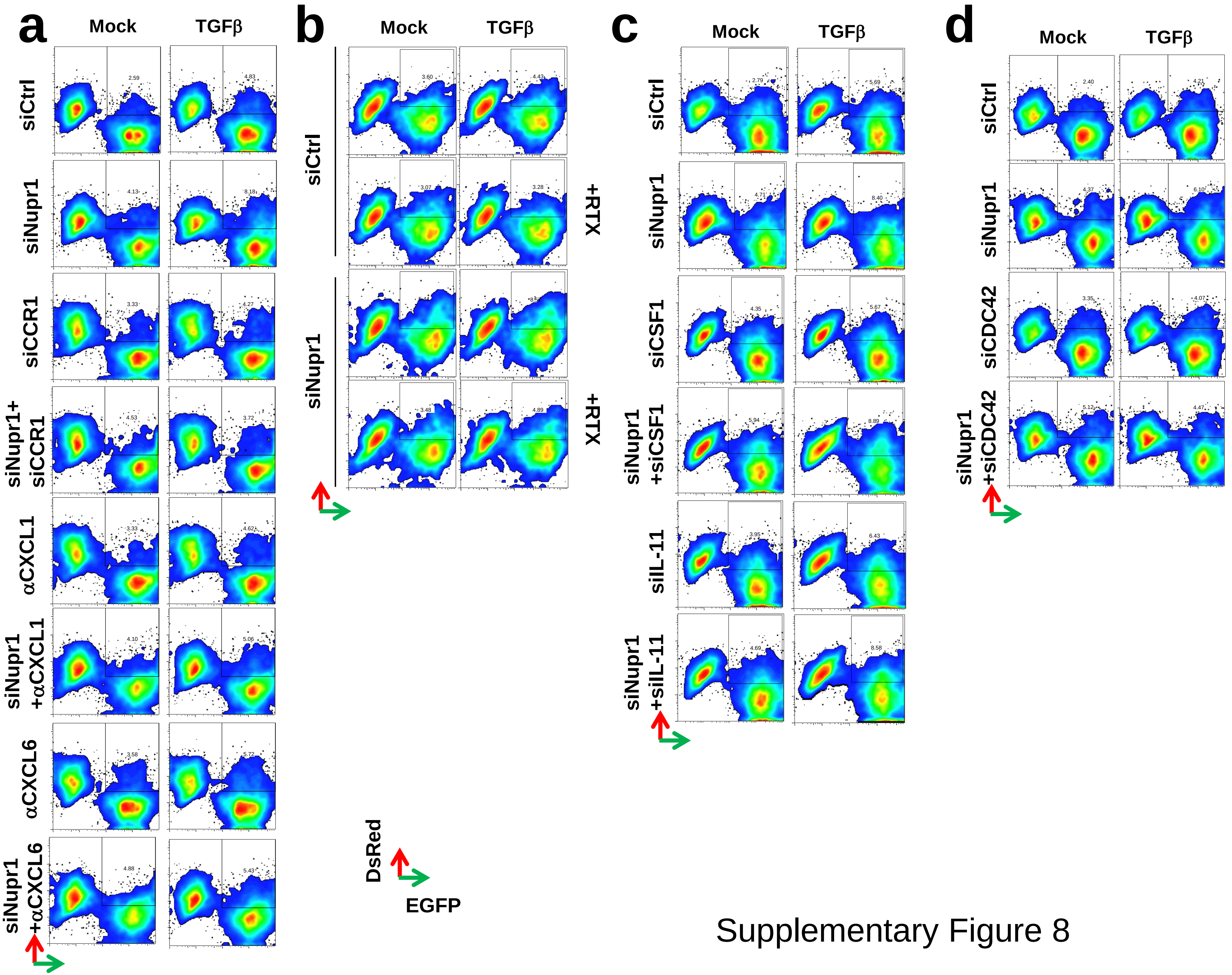

Supplementary Figure 8

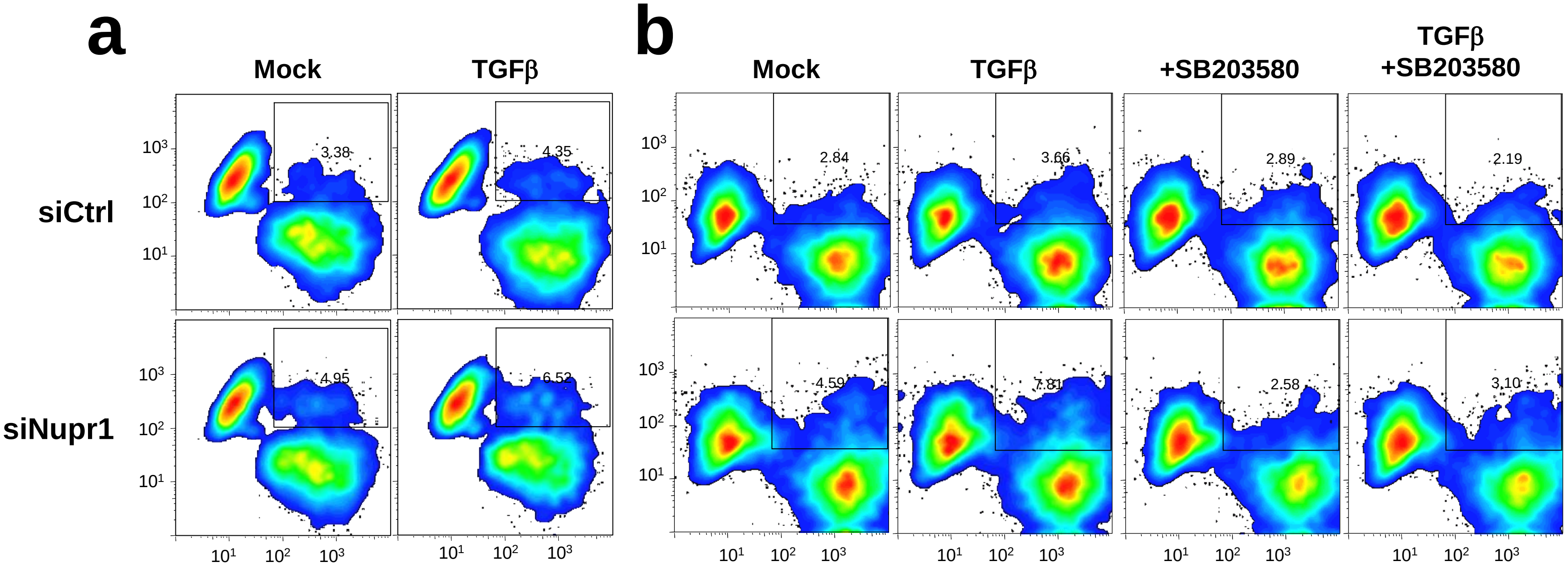

Supplementary Figure 9

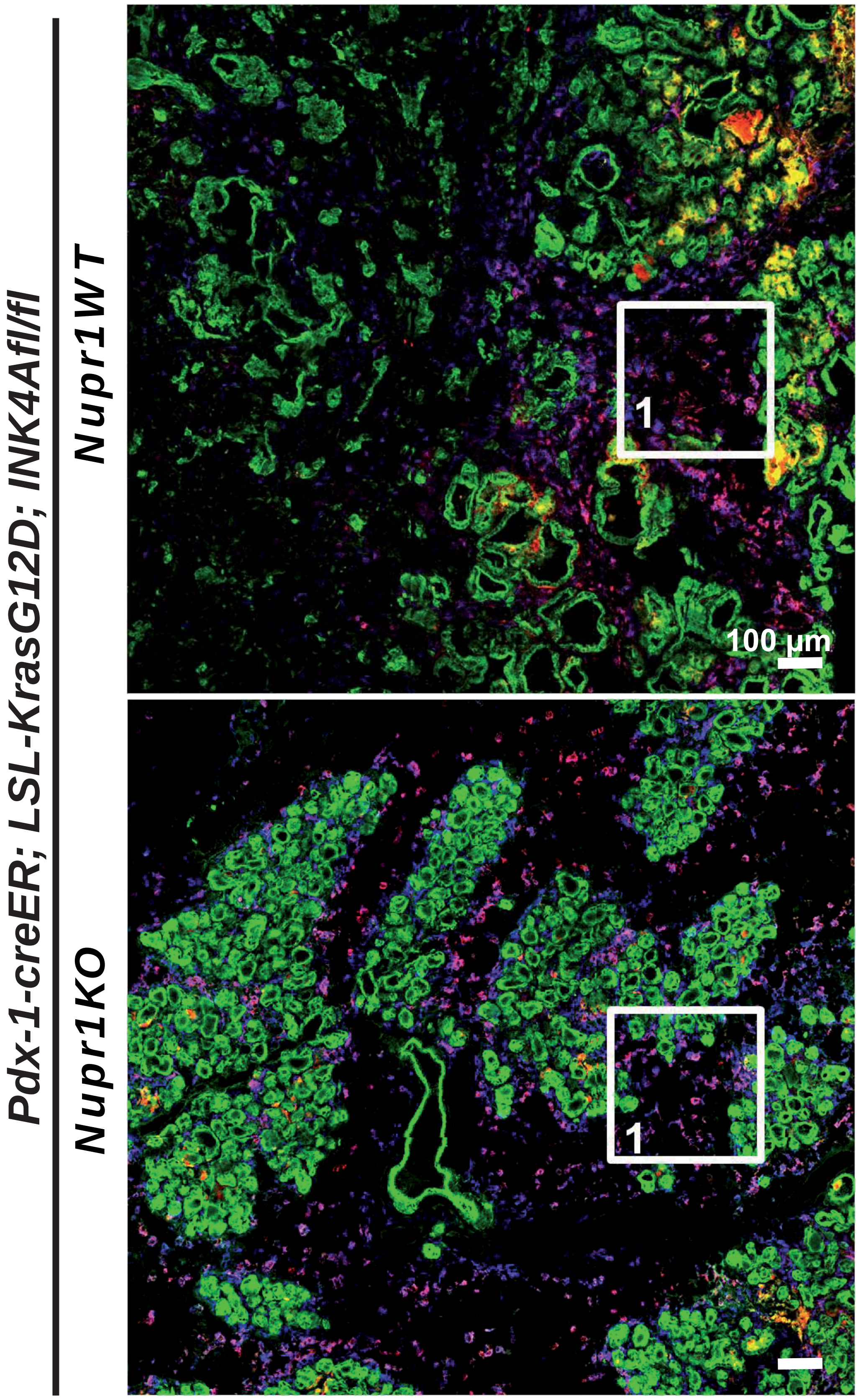

Supplementary Figure 10

Supplementary Table 1: Clinicopathological Features of Pancreatic Cancer Patients analyzed for Cell Cannibalism

PATIENT #	Cell Cannibalism	AGE	Gender	TNMR	Metastasis*	Invasion site	Chemotherapy	Radiotherapy	Post-Operatory Survival	Outcome
#01	**no**	67	M	T3N1M0R1	Yes	peritoneal carcinosis	Gemcitabine + Folfirinox©	no	109	dead
#03	**no**	69	F	T3N0M0R0	no	N/A	Gemcitabine	no	135	alive
#12	**no**	65	F	T3N1M1R1	Yes	liver	Gemcitabine	no	551	dead
#21	**no**	89	F	T3N1M0	Yes	liver	Gemcitabine	yes	389	dead
#24	**no**	74	F	T3N1M0R0	Yes	peritoneal carcinosis	Gemcitabine	yes	349	dead
#31	**no**	65	M	T3N1M0R0	no	N/A	Gemcitabine	no	99	alive
#36	**no**	77	M	T3N1M0R1	no	N/A	N/A	no	65	dead
#40	**no**	56	M	T3N1M0R0	no	N/A	Gemcitabine	no	350	alive
#48	**no**	73	F	T2N1M0	no	N/A	none	no	512	alive
#49	**no**	71	M	T3N1M0R0	Yes	liver	Gemcitabine	no	229	dead
#51	**no**	55	M	T3N0M0	Yes	liver	none	no	667	dead
#54	**no**	82	F	T3N1M0	Yes	peritoneal carcinosis	none	no	493	dead
#55	**no**	67	M	T1N1M0R0	Yes	lung	Neoadjuvant Gemcitabine + Gemcitabine	yes	119	dead
#59	**no**	75	M	T3N1M1R0	Yes	liver	Gemcitabine	no	503	dead
#61	**no**	61	M	T3N1M0R0	no	N/A	Gemcitabine	no	473	alive
#63	**no**	63	M	T3N1M0	Yes	celiac lymph node and carcinosis	none	no	398	dead
#68	**no**	67	M	T3N1M0R0	no	N/A	Gemcitabine	no	475	alive
#71	**no**	62	M	T3N1M1	Yes	peritoneal carcinosis	N/A	no	302	dead
#87	**no**	67	M	T3N1M0R0	Yes	aorta	Gemcitabine	no	607	alive
#91	**no**	64	F	T3N1bM0R0	Yes	liver	Gemcitabine	no	302	alive
#94	**no**	65	M	T3N0M0R0	no	N/A	Gemcitabine	no	61	alive
#95	**no**	58	F	T3N0M0R1	no	N/A	Gemcitabine	no	67	alive
#98	**no**	69	M	T3N1R1	Yes	peritoneal carcinosis	Gemcitabine	no	183	dead
#02	**yes**	89	M	T3N0M0R0	no	N/A	LV5FU	no	402	alive
#04	**yes**	71	F	T3N0M0R0	no	N/A	Gemcitabine	yes	553	dead
#25	**yes**	71	F	T3N0M0R0	no	N/A	Gemcitabine	no	468	alive
#26	**yes**	73	F	T3N1M0R0	Yes	liver	LV5FU 2CDDP	no	78	alive
#50	**yes**	60	F	T3N0M0R0	no	N/A	Gemcitabine	no	473	alive
#57	**yes**	69	M	T3N1M0	no	N/A	Gemcitabine	no	356	dead
#72	**yes**	75	M	T3N1M0R0	no	N/A	Gemcitabine	no	279	alive
#77	**yes**	66	M	T3N1bM0R0	Yes	liver	Neoadjuvant Gemcitabine + Folfirinox©	no	88	alive
#80	**yes**	53	M	T4N1M0R0	no	N/A	N/A	no	98	dead
#87	**yes**	81	F	T3N0M0R0	no	N/A	Gemcitabine	no	306	alive

#88	**yes**	80	M	T3N0M0R0	no	N/A	Gemcitabine	no	186	alive
#92	**yes**	68	F	T3N0M0	no	N/A	LV5FU[2] + 2CDDP[3]	yes	424	alive
#60	**yes**	86	H	T3N0M0R0	no	N/A	Gemcitabine	no	268	dead

*** TNM Classification (UICC 2009) : T(Tumor) :T1=<2 cm in situ ; T2=>2 cm in situ ; T3= peri-pancreatic tissue invasion without invasion of the celiac axis or the mesenteric artery ; T4= celiac or mesenteric artery invasion. N(Nodal involvement): N0= none, N1= one lymph node involved, N1b =multiple nodal invasion. M(distal metastasis): M0= none, M1= distal metastasis, Mx= insufficient information. R(resection edges):R0= complete resection, R1= incomplete resection with microscopical residues . [2] LV5FU: Calcium Levofolinate + 5-Fluorouracil. [3] 2CDDP: Cisplatin.**

Supplementary Table 2 : Myeloid- and phagocytosis-related genes modulated by Nupr1 in Panc-1 cells upon TGFβ stimulation.

AFFY_ID	Symbol	Name	CC_siRNAc_Mock.cel*	CC_siRNAc_TGFb_7H.cel*	CC_siRNAc_TGFb_24H.cel*	CC_siRNANupr1_Mock.cel*	CC_siRNA Nupr1_TGFb_7H.cel*	CC_siRNA Nupr1_TGFb_24H.cel*	P.Value	Biological Process (GO)
201110_s_at	THBS1	thrombospondin 1	73	81	114	111	190	181	0.02976828	7275 // development // traceable author statement /// 6928 // cell motility // not recorded /// 7155 // cell adhesion // inferred from electronic annotation /// 7596 // blood coagulation // not recorded /// 7399 // neurogenesis // not recorded
201487_at	CTSC	cathepsin C	1575	1672	1863	1985	2179	2436	0.042933702	6508 // proteolysis and peptidolysis // not recorded /// 6955 // immune response // traceable author statement
209682_at	CBLB	Cas-Br-M (murine) ecotropic retroviral transforming sequence b	199	228	267	321	340	402	0.01006776	4871 // signal transducer activity // extended: inferred from electronic annotation
213012_at	NEDD4	neural precursor cell expressed, developmentally down-regulated 4	295	399	529	614	659	723	0.022159911	6512 // ubiquitin cycle // inferred from electronic annotation
210136_at	MBP	myelin basic protein	1154	1027	1157	2341	2005	1619	0.002845908	7268 // synaptic transmission // traceable author statement /// 7417 // central nervous system development // traceable author statement /// 6955 // immune response // traceable author statement
202541_at	SCYE1	small inducible cytokine subfamily E, member 1 (endothelial monocyte-activating)	412	467	598	834	860	812	0.003429095	6954 // inflammatory response // traceable author statement /// 6418 // amino acid activation // traceable author statement /// 6935 // chemotaxis // traceable author statement
235255_at	ATP6V0A2	ATPase, H+ transporting, lysosomal V0 subunit a2	64	67	65	142	135	118	0.000219553	6955 // immune response // experimental evidence /// 15992 // proton transport
217990_at	GMPR2	guanosine monophosphate reductase 2	899	834	900	1441	1470	1345	0.000719249	9117 // nucleotide metabolism // inferred from electronic annotation
207594_s_at	SYNJ1	synaptojanin 1	60	86	81	115	125	108	0.013285462	8099 // synaptic vesicle endocytosis // predicted/computed /// 6796 // phosphate metabolism // predicted/computed /// 6899 // nonselective vesicle transport // predicted/computed
208979_at	NCOA6	nuclear receptor coactivator 6	462	400	447	750	710	726	0.000670827	6355 // regulation of transcription, DNA-dependent // inferred from electronic annotation /// 7507 // heart development // inferred from sequence or structural similarity /// 7420 // brain development // inferred from sequence or structural similarity /// 6367 // transcription initiation from Pol II promoter // inferred from sequence or structural similarity /// 1701 // embryonic development (sensu Mammalia) // inferred from sequence or structural similarity /// 30099 // myeloid blood cell differentiation // inferred from direct assay
204035_at	SCG2	secretogranin II (chromogranin C)	36	27	42	143	104	103	0.000319735	9306 // protein secretion // traceable author statement
208405_s_at	CD164	CD164 molecule, sialomucin	4230	4482	5194	8596	8022	7953	0.000682767	6955 // immune response // predicted/computed /// 7165 // signal transduction // predicted/computed /// 7275 // development // predicted/computed /// 7155 // cell adhesion // experimental evidence /// 8285 // negative regulation of cell proliferation // predicted/computed
213168_at	SP3	Sp3 transcription factor	1441	1490	1478	1881	1847	1896	0.012824293	6355 // regulation of transcription, DNA-dependent // non-traceable author statement

* Actual RMA normalized data from the array

209481_at	SNRK	SNF related kinase	284	280	319	374	362	358	0.037401277	6468 // protein amino acid phosphorylation // inferred from electronic annotation
203381_s_at	APOE	apolipoprotein E	2657	2575	2933	3573	3758	3851	0.006737008	6955 // immune response // not recorded /// 6629 // lipid metabolism // not recorded /// 8015 // circulation // traceable author statement /// 7275 // development // not recorded /// 6869 // lipid transport // inferred from electronic annotation /// 8203 // cholesterol metabolism // traceable author statement
202948_at	IL1R1	interleukin 1 receptor, type I	231	237	290	390	407	462	0.002340548	7166 // cell surface receptor linked signal transduction // traceable author statement /// 6954 // inflammatory response // not recorded /// 6955 // immune response // traceable author statement
210232_at	CDC42	cell division cycle 42 (GTP binding protein, 25kDa)	44	52	57	164	133	190	0.00015176	7015 // actin filament organization // non-traceable author statement /// 7264 // small GTPase mediated signal transduction // inferred from electronic annotation
203187_at	DOCK1	dedicator of cytokinesis 1	223	241	293	367	430	389	0.004867063	7165 // signal transduction // traceable author statement /// 7229 // integrin-mediated signaling pathway // traceable author statement /// 7264 // small GTPase mediated signal transduction // traceable author statement /// 6915 // apoptosis // traceable author statement /// 6911 // phagocytosis, engulfment // traceable author statement
211013_x_at	PML	promyelocytic leukemia	362	342	330	421	514	503	0.011330197	7048 // oncogenesis // experimental evidence
215116_s_at	DNM1	dynamin 1	133	149	139	298	405	404	0.0002007	6898 // receptor mediated endocytosis // traceable author statement /// 7268 // synaptic transmission // not recorded
210223_s_at	MR1	major histocompatibility complex, class I-related	150	138	124	198	222	211	0.002607999	6955 // immune response // predicted/computed
35776_at	ITSN1	intersectin 1 (SH3 domain protein)	323	350	370	455	502	456	0.009513646	6897 // endocytosis // inferred from electronic annotation
224909_s_at	PREX1	phosphatidylinositol 3,4,5-trisphosphate-dependent RAC exchanger 1	283	341	347	570	722	602	0.000802582	42119 // neutrophil activation // traceable author statement /// 6801 // superoxide metabolism // traceable author statement /// 7242 // intracellular signaling cascade // inferred from electronic annotation /// 30041 // actin filament polymerization // traceable author statement
216627_s_at	B4GALT1	UDP-Gal:betaGlcNAc beta 1,4- galactosyltransferase, polypeptide 1	81	109	114	170	301	250	0.003583858	---
235287_at	CDK6	cyclin-dependent kinase 6	105	116	126	188	228	201	0.000900294	74 // regulation of cell cycle // traceable author statement /// 80 // G1 phase of mitotic cell cycle // traceable author statement /// 910 // cytokinesis // inferred from electronic annotation /// 6468 // protein amino acid phosphorylation // inferred from electronic annotation
223465_at	COL4A3BP	collagen, type IV, alpha 3 (Goodpasture antigen) binding protein	137	168	183	285	307	237	0.004390721	6955 // immune response // non-traceable author statement /// 6468 // protein amino acid phosphorylation // traceable author statement
226547_at	MYST3	MYST histone acetyltransferase (monocytic leukemia) 3	198	227	259	357	340	315	0.007527693	6355 // regulation of transcription, DNA-dependent // inferred from electronic annotation /// 6323 // DNA packaging // traceable author statement /// 6334 // nucleosome assembly // inferred from electronic annotation /// 8151 // cell growth and/or maintenance // inferred from electronic annotation
203507_at	CD68	CD68 molecule	127	132	120	137	149	160	0.128801885	5624 // membrane fraction // traceable author statement /// 16021 // integral to membrane // traceable author statement /// 16020 // membrane // extended:Unknown; Lamp; 7.5e-182 /// 5764 // lysosome // inferred from electronic annotation
206336_at	CXCL6	chemokine (C-X-C motif) ligand 6 (granulocyte chemotactic protein 2)	15	17	16	20	28	33	0.009567931	6954 // inflammatory response // experimental evidence /// 6935 // chemotaxis // experimental evidence /// 7165 // signal transduction // predicted/computed /// 7267 // cell-cell signaling // predicted/computed
204470_at	CXCL1	chemokine (C-X-C motif) ligand 1 (melanoma growth stimulating activity, alpha)	112	149	124	171	255	198	0.015386183	6954 // inflammatory response // traceable author statement /// 7186 // G-protein coupled receptor protein signaling pathway // traceable author statement /// 30036 // actin cytoskeleton organization and biogenesis // traceable author statement /// 6935 // chemotaxis // traceable author statement /// 7401 // pan-neural process // traceable author statement /// 8283 // cell proliferation // traceable author statement /// 6955 // immune response // inferred from electronic annotation /// 7242 // intracellular signaling cascade // traceable author statement /// 8285 // negative regulation of cell proliferation // traceable author statement
205558_at	TRAF6	TNF receptor-associated factor 6	332	407	333	403	416	408	0.234230806	7165 // signal transduction // traceable author statement
210805_x_at	RUNX1	runt-related transcription factor 1 (acute myeloid leukemia 1; aml1 oncogene)	29	28	33	55	56	61	0.000349187	6355 // regulation of transcription, DNA-dependent // non-traceable author statement /// 7275 // development // traceable author statement /// 8151 // cell growth and/or maintenance // inferred from electronic annotation
214575_s_at	AZU1	azurocidin 1 (cationic antimicrobial protein 37)	72	70	61	104	111	83	0.013358766	6805 // xenobiotic metabolism // inferred from electronic annotation /// 6935 // chemotaxis // not recorded /// 6508 // proteolysis and peptidolysis // inferred from electronic annotation

207082_at	CSF1	colony stimulating factor 1 (macrophage)	151	128	93	250	254	167	0.018536766	30225 // macrophage differentiation // traceable author statement /// 8283 // cell proliferation // non-traceable author statement /// 6960 // antimicrobial humoral response (sensu Invertebrata) // not recorded /// 8284 // positive regulation of cell proliferation // traceable author statement /// 30097 // hemopoiesis // non-traceable author statement /// 30154 // cell differentiation // non-traceable author statement
209558_s_at	HIP1R	huntingtin interacting protein 1 related	197	192	175	215	234	215	0.114671144	---
200991_s_at	SNX17	sorting nexin 17	3225	2808	2558	3205	3225	3096	0.379041867	30100 // regulation of endocytosis // non-traceable author statement /// 6886 // intracellular protein transport // non-traceable author statement /// 16197 // endosome transport // non-traceable author statement /// 45192 // low-density lipoprotein catabolism // non-traceable author statement /// 7242 // intracellular signaling cascade // inferred from electronic annotation /// 7218 // neuropeptide signaling pathway // inferred from electronic annotation
232331_at	PPARG	peroxisome proliferator-activated receptor gamma	220	239	213	135	138	116	0.000794543	---
205731_s_at	NCOA2	nuclear receptor coactivator 2	133	149	147	178	167	165	0.080708343	6355 // regulation of transcription, DNA-dependent // non-traceable author statement /// 7165 // signal transduction // inferred from electronic annotation
203085_s_at	**TGFB1**	**transforming growth factor, beta 1**	416	557	565	541	933	1046	0.083198485	8283 // cell proliferation // inferred from electronic annotation /// 6916 // anti-apoptosis // traceable author statement /// 7267 // cell-cell signaling // not recorded /// 16049 // cell growth // inferred from electronic annotation /// 7179 // TGFbeta receptor signaling pathway // not recorded /// 74 // regulation of cell cycle // inferred from electronic annotation /// 40007 // growth // inferred from electronic annotation
206924_at	IL11	interleukin 11	88	141	190	105	282	464	0.298975642	7267 // cell-cell signaling // Unknown /// 8284 // positive regulation of cell proliferation // inferred from direct assay /// 45444 // adipocyte differentiation // non-traceable author statement /// 8283 // cell proliferation // traceable author statement /// 30219 // megakaryocyte differentiation // non-traceable author statement /// 30183 // B-cell differentiation // non-traceable author statement /// 30168 // platelet activation // non-traceable author statement
209083_at	CORO1A	coronin, actin binding protein, 1A	144	142	138	130	216	168	0.387528279	6912 // phagosome formation // not recorded /// 7067 // mitosis // not recorded /// 6928 // cell motility // not recorded /// 6810 // transport // not recorded
239796_x_at	TIRAP	toll-interleukin 1 receptor (TIR) domain containing adaptor protein	153	138	150	125	122	100	0.054755122	---
202430_s_at	PLSCR1	phospholipid scramblase 1	153	124	153	124	105	105	0.061787005	30168 // platelet activation // non-traceable author statement /// 17121 // phospholipid scrambling // traceable author statement
204039_at	CEBPA	CCAAT/enhancer binding protein (C/EBP), alpha	108	107	79	69	60	67	0.015704743	---
1552256_a_at	SCARB1	scavenger receptor class B, member 1	2037	2263	1954	1572	1830	1385	0.047767136	7155 // cell adhesion // extended:inferred from sequence similarity; CD36; 2e-239
200901_s_at	M6PR	mannose-6-phosphate receptor (cation dependent)	1459	1454	1510	534	797	708	0.000701576	6898 // receptor mediated endocytosis // traceable author statement /// 8333 // endosome to lysosome transport // traceable author statement /// 6810 // transport // inferred from electronic annotation
213373_s_at	CASP8	caspase 8, apoptosis-related cysteine peptidase	934	916	942	635	770	712	0.01641571	6508 // proteolysis and peptidolysis // traceable author statement /// 8632 // apoptotic program // traceable author statement
206788_s_at	CBFB	core-binding factor, beta subunit	983	1768	1757	784	1256	1018	0.183335483	6366 // transcription from Pol II promoter // predicted/computed /// 7048 // oncogenesis // predicted/computed

Supplementary Table 3 : GO-ANOVA Analysis.

GO ID	GO Description	p-value(Nupr1status)	p-value(siCtrl vs. siNupr1)	Ratio(siCtrl vs. siNupr1)	Fold-Change(siCtrl vs. siNupr1)	Fold-Change(siCtrl vs. siNupr1) (Description)	F(Nupr1status)	F(Marker ID)	F(Sample ID(Nupr1status))	SS(Error)	F(Error)
2761	regulation of myeloid leukocyte differentiation	0,000629138	0,000629138	0,91978	-1,08722	siCtrl down vs siNupr1	94,3322	312,866	0,268062	28,6713	1
2274	myeloid leukocyte activation	0,00214641	0,00214641	0,893518	-1,11917	siCtrl down vs siNupr1	49,554	480,566	0,389795	8,31837	1
30099	myeloid cell differentiation	0,0114085	0,0114085	0,937862	-1,06626	siCtrl down vs siNupr1	19,6384	536,121	0,553749	26,1242	1
2573	myeloid leukocyte differentiation	0,012251	0,012251	0,861783	-1,16039	siCtrl down vs siNupr1	18,8373	503,481	1,05311	10,4471	1
2444	myeloid leukocyte mediated immunity	0,0123983	0,0123983	0,826785	-1,2095	siCtrl down vs siNupr1	18,7057	695,459	1,22014	1,78168	1

Gene Symbol	Gene Title	RefSeq Transcript ID	p-value (Nupr1status)	p-value(siCtrl vs. siNupr1)	qvalue(p-value(siCtrl vs. siNupr1))	qvalue(p-value(siCtrl vs. siNupr1))	Ratio(siCtrl vs. siNupr1)	Fold-Change (siCtrl vs. siNupr1)	Fold-Change(siCtrl vs. siNupr1) (Description)	F(Nupr1status)	SS(Nupr1status)	SS(Error)	F(Error)
PPARA	peroxisome proliferator-activated receptor alpha	NM_001001928 /// NM_005036	0,000375756	0,00037576	0,0170633	0,0170598	0,427624	-2,3385	siCtrl down vs siNupr1	123,037	2,25308	0,07325	1
RUNX1	runt-related transcription factor 1	NM_001001890 /// NM_001122607 /// NM_001754	0,000476419	0,00047642	0,0182843	0,0182805	0,5282	-1,89322	siCtrl down vs siNupr1	108,897	1,27193	0,04672	1
NR1H3	nuclear receptor subfamily 1, group H, member 3	NM_001130101 /// NM_001130102 /// NM_005693	0,000516804	0,0005168	0,0187915	0,0187876	0,659966	-1,51523	siCtrl down vs siNupr1	104,423	0,539165	0,02065	1
ABCA1	ATP-binding cassette, sub-family A (ABC1), member 1	NM_005502	0,000653146	0,00065315	0,0199516	0,0199475	0,490289	-2,03961	siCtrl down vs siNupr1	92,5207	1,58609	0,06857	1
NFKBIA	nuclear factor of kappa light polypeptide gene enhancer in B-cells inhibitor, al	NM_020529	0,00147496	0,00147496	0,0261202	0,0261149	2,09383	2,09383	siCtrl up vs siNupr1	60,4602	1,705	0,1128	1
ACIN1	apoptotic chromatin condensation inducer 1	NM_001164814 /// NM_001164815 /// NM_001164816 /// NM_001164817 /// NM_014977	0,00246383	0,00246383	0,031182	0,0311756	0,73843	-1,35422	siCtrl down vs siNupr1	46,0322	0,287066	0,02494	1
ZNF675	zinc finger protein 675	NM_138330	0,00266852	0,00266852	0,0322383	0,0322316	0,563618	-1,77425	siCtrl down vs siNupr1	44,1025	1,02642	0,09309	1
CALCA	calcitonin-related polypeptide alpha	NM_001033952 /// NM_001033953 /// NM_001741	0,0103372	0,0103372	0,0601059	0,0600934	0,942847	-1,06062	siCtrl down vs siNupr1	20,7955	0,0108133	0,00208	1
AGTR1	angiotensin II receptor, type 1	NM_000685 /// NM_004835 /// NM_009585 /// NM_031850 /// NM_032049	0,0129559	0,0129559	0,0670306	0,0670167	0,807384	-1,23857	siCtrl down vs siNupr1	18,2281	0,142918	0,03136	1

Supplementary Table 3 : GO-ANOVA Analysis.

NFKB1	nuclear factor of kappa light polypeptide gene enhancer in B-cells 1	NM_001165412 /// NM_003998	0,0130523	0,0130523	0,0672521	0,0672385	1,36778	1,36778	siCtrl up vs siNupr1	18,1487	0,306237	0,0675	1
CARTPT	CART prepropeptide	NM_004291	0,0137527	0,0137527	0,0688073	0,0687932	1,05732	1,05732	siCtrl up vs siNupr1	17,5968	0,00969961	0,0022	1
ADIPOQ	adiponectin, C1Q and collagen domain containing	NM_001177800 /// NM_004797	0,0228922	0,0228922	0,0887802	0,0887619	1,30083	1,30083	siCtrl up vs siNupr1	12,9126	0,215956	0,0669	1
PLA2G10	phospholipase A2, group X	NM_003561	0,0256958	0,0256958	0,0939976	0,0939782	1,18972	1,18972	siCtrl up vs siNupr1	12,0076	0,0942157	0,03139	1
ITGB3	integrin, beta 3 (platelet glycoprotein IIIa, antigen CD61)	NM_000212	0,0285736	0,0285736	0,0992617	0,0992415	0,658642	-1,51828	siCtrl down vs siNupr1	11,2213	0,544389	0,19406	1
BGLAP /// PMF1	bone gamma-carboxyglutamate (gla) protein /// polyamine-modulated factor 1	NM_199173	0,0315904	0,0315904	0,104839	0,104818	0,817965	-1,22255	siCtrl down vs siNupr1	10,5157	0,126053	0,04795	1
NR1H2	nuclear receptor subfamily 1, group H, member 2	NM_007121	0,0325552	0,0325552	0,10623	0,106208	0,767208	-1,30343	siCtrl down vs siNupr1	10,3111	0,219242	0,08505	1
CSF1	colony stimulating factor 1 (macrophage)	NM_000757 /// NM_172210 /// NM_172211 /// NM_172212	0,0397001	0,0397001	0,118767	0,118742	0,551724	-1,8125	siCtrl down vs siNupr1	9,03702	1,1042	0,48874	1

Supplementary Table 4: EMT- related genes modulated by Nupr1 in Panc-1 cells upon TGFβ stimulation.

Affy ID	Symbol	Name	P.Value	CC_siRNAc_Mock.cel	CC_siRNAc_TGFb_7H.cel	CC_siRNAc_TGFb_24H.cel	CC_siRNA Nupr1_Mock.cel	CC_siRNA Nupr1_TGFb_7H.cel	CC_siRNA Nupr1_TGFb_24H.cel	Biological Process (GO)
243140_at	ACTA2	actin, alpha 2, smooth muscle, aorta	0.28090717	16	16	14	14	13	14	7517 // muscle development // non-traceable author statement
203324_s_at	CAV2	caveolin 2	0.00881085	3828	3878	4208	5454	5240	5125	---
201131_s_at	CDH1	cadherin 1, type 1, E-cadherin (epithelial)	0.50898017	701	644	329	467	553	246	---
203440_at	CDH2	cadherin 2, type 1, N-cadherin (neuronal)	0.09755644	106	109	93	83	80	92	7156 // homophilic cell adhesion // inferred from electronic annotation /// 7155 // cell adhesion // traceable author statement
218182_s_at	CLDN1	claudin 1	0.0655784	205	194	192	371	346	207	7155 // cell adhesion // traceable author statement
203954_x_at	CLDN3	claudin 3	0.42939301	285	199	181	216	185	150	---
202790_at	CLDN7	claudin 7	0.00326471	701	551	592	1119	1056	891	---
1554411_at	CTNNB1	catenin (cadherin-associated protein), beta 1, 88kDa	0.02153537	75	73	81	62	59	61	6350 // transcription // inferred from electronic annotation /// 7155 // cell adhesion // inferred from electronic annotation /// 6357 // regulation of transcription from Pol II promoter // not recorded
227561_at	DDR2	discoidin domain receptor tyrosine kinase 2	0.00060277	116	160	121	284	262	291	7165 // signal transduction // traceable author statement /// 7155 // cell adhesion // traceable author statement /// 7169 // transmembrane receptor protein tyrosine kinase signaling pathway // inferred from electronic annotation /// 6468 // protein amino acid phosphorylation // inferred from electronic annotation
218995_s_at	EDN1	endothelin 1	0.01883358	795	1134	949	554	727	589	8217 // regulation of blood pressure // not recorded /// 7165 // signal transduction // traceable author statement /// 7267 // cell-cell signaling // not recorded /// 8284 // positive regulation of cell proliferation // traceable author statement /// 9405 // pathogenesis // inferred from electronic annotation
208229_at	FGFR2	fibroblast growth factor receptor 2 (bacteria-expressed kinase, keratinocyte growth factor receptor, craniofacial dysostosis 1, Crouzon syndrome, Pfeiffer syndrome, Jackson-Weiss syndrome)	0.14159658	103	103	80	72	88	69	---
203689_s_at	FMR1	fragile X mental retardation 1	0.14555935	1217	1338	1415	1107	1195	1099	---
214520_at	FOXC2	forkhead box C2 (MFH-1, mesenchyme forkhead 1)	0.28417847	86	68	65	57	67	64	6355 // regulation of transcription, DNA-dependent // inferred from electronic annotation /// 7498 // mesoderm development // traceable author statement /// 6366 // transcription from Pol II promoter // traceable author statement
202351_at	ITGAV	integrin, alpha V (vitronectin receptor, alpha polypeptide, antigen CD51)	0.2303204	4002	5000	5928	2927	3834	4753	7160 // cell-matrix adhesion // inferred from electronic annotation /// 7229 // integrin-mediated signaling pathway // inferred from electronic annotation
228491_at	KRT19	keratin 19	0.3559775	19909	17659	16383	19805	19014	16576	---
221558_s_at	LEF1	lymphoid enhancer-binding factor 1	0.00037419	204	202	189	112	110	94	6355 // regulation of transcription, DNA-dependent // inferred from electronic annotation
207233_s_at	MITF	microphthalmia-associated transcription factor	0.0526219	387	344	408	536	484	435	---
1566678_at	MMP2	matrix metallopeptidase 2 (gelatinase A, 72kDa gelatinase, 72kDa type IV collagenase)	0.23796425	57	60	55	49	52	51	30574 // collagen catabolism // inferred from electronic annotation

207847_s_at	MUC1	mucin 1, cell surface associated	0.03422693	101	105	86	148	153	112	---
209925_at	OCLN	occludin	0.70430591	1568	1456	1269	2070	1429	1280	6461 // protein complex assembly // traceable author statement
203186_s_at	S100A4	S100 calcium binding protein A4	0.46173294	12309	12408	12283	11815	11575	11034	7125 // invasive growth // not recorded
1568765_at	SERPINE 1	serpin peptidase inhibitor, clade E (nexin, plasminogen activator inhibitor type 1), member 1	0.56620425	114	268	258	87	200	193	7596 // blood coagulation // traceable author statement
211114_x_at	SIP1	survival of motor neuron protein interacting protein 1	0.01648116	292	309	324	452	407	377	245 // spliceosome assembly // experimental evidence /// 6397 // mRNA processing // experimental evidence
1556154_a_at	SNAI3	snail homolog 3 (Drosophila)	0.06529162	38	38	35	48	42	46	---
202935_s_at	SOX9	SRY (sex determining region Y)-box 9 (campomelic dysplasia, autosomal sex-reversal)	0.01201282	3012	2873	2979	2230	2248	2386	1502 // cartilage condensation // not recorded /// 6357 // regulation of transcription from Pol II promoter // traceable author statement
225289_at	STAT3	signal transducer and activator of transcription 3 (acute-phase response factor)	0.01747467	773	649	671	992	941	869	---
240188_at	TGFBR3	transforming growth factor, beta receptor III	0.15544694	61	65	63	59	51	52	---
229404_at	TWIST2	twist homolog 2 (Drosophila)	0.1491382	298	229	240	230	183	206	45668 // negative regulation of osteoblast differentiation // inferred from direct assay /// 30154 // cell differentiation // inferred from electronic annotation /// 45892 // negative regulation of transcription, DNA-dependent // inferred from sequence or structural similarity
223642_at	ZIC2	Zic family member 2 (odd-paired homolog, Drosophila)	0.04528176	1118	857	898	821	600	625	7420 // brain development // traceable author statement
